# Bone strength and composition in spacefaring rodents: systematic review and meta-analysis

**DOI:** 10.1038/s41526-022-00195-7

**Published:** 2022-04-13

**Authors:** Matthew Goldsmith, Sequoia D. Crooks, Sean F. Condon, Bettina M. Willie, Svetlana V. Komarova

**Affiliations:** 1 Research Centre, Shriners Hospital for Children – Canada, Montréal, QC Canada; 2 Faculty of Dental Medicine and Oral Health Sciences, McGill University, Montréal, QC Canada; 3 Department of Pediatric Surgery, McGill University, Montréal, QC Canada

**Keywords:** Physiology, Metabolic bone disease

## Abstract

Studying the effects of space travel on bone of experimental animals provides unique advantages, including the ability to perform post-mortem analysis and mechanical testing. To synthesize the available data to assess how much and how consistently bone strength and composition parameters are affected by spaceflight, we systematically identified studies reporting bone health in spacefaring animals from Medline, Embase, Web of Science, BIOSIS, and NASA Technical reports. Previously, we reported the effect of spaceflight on bone architecture and turnover in rodents and primates. For this study, we selected 28 articles reporting bone strength and composition in 60 rats and 60 mice from 17 space missions ranging from 7 to 33 days in duration. Whole bone mechanical indices were significantly decreased in spaceflight rodents, with the percent difference between spaceflight and ground control animals for maximum load of −15.24% [Confidence interval: −22.32, −8.17]. Bone mineral density and calcium content were significantly decreased in spaceflight rodents by −3.13% [−4.96, −1.29] and −1.75% [−2.97, −0.52] respectively. Thus, large deficits in bone architecture (6% loss in cortical area identified in a previous study) as well as changes in bone mass and tissue composition likely lead to bone strength reduction in spaceflight animals.

## Introduction

Long-duration spaceflight is now firmly on the agenda for humanity^1^. Currently, with plans for a human-manned mission to the Martian surface within the next two decades^2^ and plans for the construction of a lunar outpost to facilitate deep-space exploration^3^, we can expect that in the coming century human spaceflights will increase in frequency and duration. Longer space missions pose greater risk to human health, potentially augmenting the known spaceflight related physiological changes including bone loss^4–6^. Although countermeasures have been implemented to help mitigate microgravity-induced bone loss – primarily exercise & diet^4,5^ – they have not been completely effective^5,7^. To enable development of countermeasures that prevent microgravity-induced bone loss, comprehensive understanding of the underlying phenomena is necesary^4,5^.

Animals have long been used as a model to study and understand physiological changes that result from various stimuli in humans. Specifically in regards to microgravity and bone, animal studies have the benefit of post-mortem analysis, which enabled bone mechanical testing to be performed on spaceflight subjects. This allows for direct measurement of bone strength, and thus more accurate assessment of fracture risk. Bone strength is determined by various contributors including bone geometry, bone mass, and the properties of the constituent tissue^8–10^. In humans, direct measurement of bone strength is not possible, and one must rely on surrogate measures such as bone mineral density measured through clinical imaging (i.e., dual-energy X-ray absorptiometry, DEXA, or peripheral quantitative computed tomography, pQCT) or estimation of strength using finite element analyses to predict fracture risk ^9,11]^. Thus, animal experiments can be used to better understand changes occurring in humans during long-duration missions^12^.

Meta-analysis is an important approach for quantitative synthesis of prior work, especially for spaceflight experiments, which are tremendously expensive and have small sample size, making improved statistical power with meta-analysis very important. Moreover, summarizing all the missions that occurred in different crafts that flew to space over 40–50 years, allows to separate the common effects of spaceflight from hazards and potential mishaps occurring within individual missions. The current study serves as a continuation of our team’s series of systematic reviews and meta-analyses regarding spaceflight-induced changes to bone in humans^6^ and animals^13^. Previously, we demonstrated a significant deterioration of both cortical and trabecular bone architecture in spaceflight rodents and found bone turnover to be significantly affected^13^. Here, we analyzed the data reporting changes to bone mechanical properties, bone mass, characterized by bone tissue mineral density (BMD) and bone composition in spaceflight animals. The goals of the present study were to (*i*) to systematically identify all available literature concerning the mechanical properties, BMD and composition of bone in animals sent to space; (*ii*) to quantitatively characterize the degree and consistency of change in bone strength and composition parameters using a meta-analytic approach, and (*iii*) identify confounding variables associated with observed changes to the included bone parameters. Analyzing how bone strength and composition are affected by spaceflight will provide further insights into the underlying causes and the functional risks microgravity can pose to humans.

## Results

### Identified articles

**Fig. 1:**
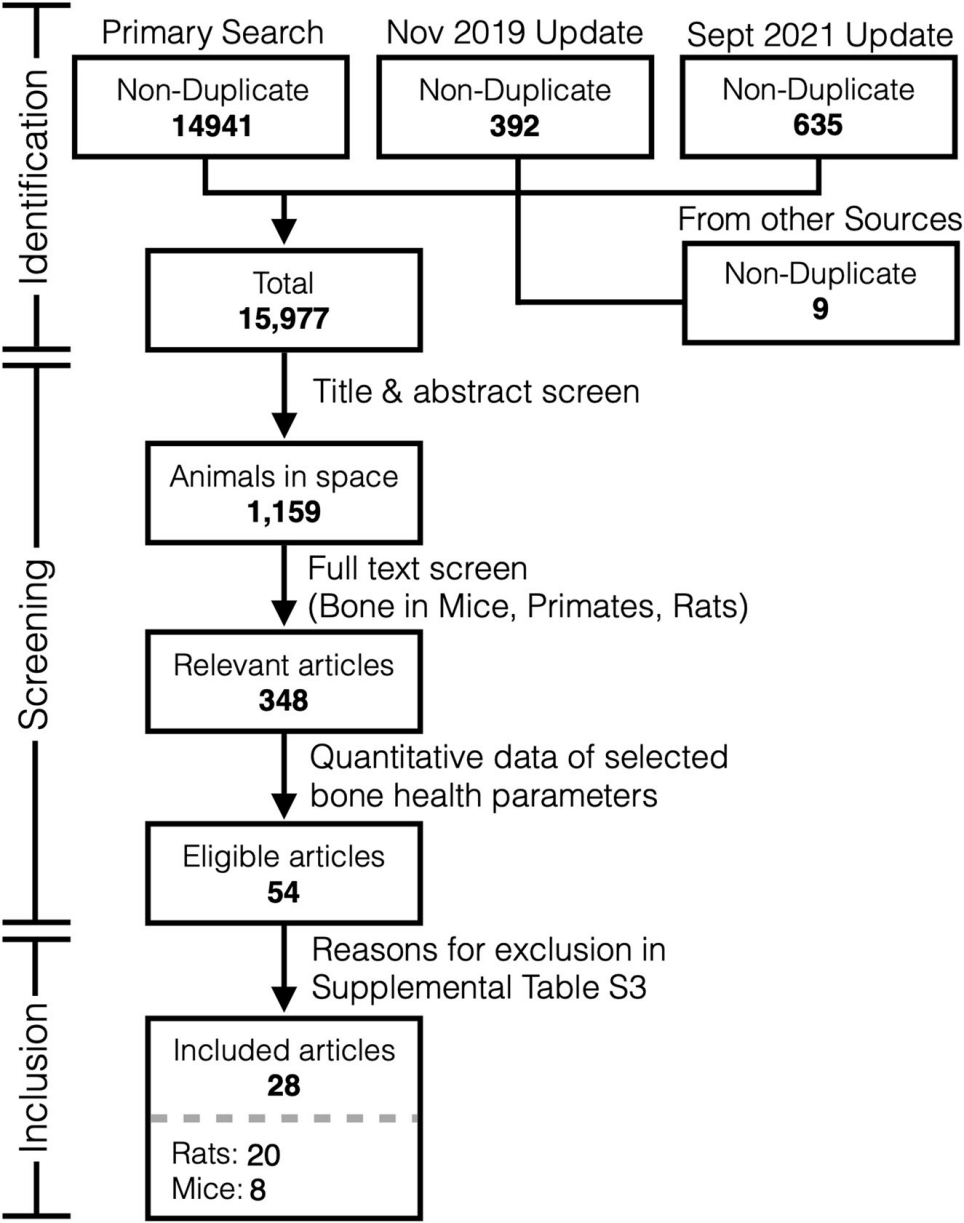
Systematic review information flow. Prisma diagram indicated the numbers of records assessed duringdifferent steps of the systematic review.

The systematic searches were performed on Medline, Embase, PubMed, BIOSIS Previews, and Web of Science using the search strategy reported in Fu, Goldsmith et al.^13^. Additionally, 9 articles were identified from other sources including the NASA Technical Reporting Service and articles referenced in the compendium of NASA’s animal and cell spaceflight experiments compiled by Ronca et al^12^. Original search was performed on November 2, 2017, a full update was performed on November 1, 2019 and again on September 13, 2021. In total, 15,977 candidate non-duplicate articles were identified (Fig. 1). The Preferred Reporting Items for Systematic Reviews and Meta-Analysis (PRISMA) checklist is provided in the Supplementary Table 1. Following title and abstract screening, 1159 were determined to be concerning animals sent to space. Previously, we identified that a majority of bone health-focused animal studies reported findings in mice, rats and primates (348 articles)^13^. In this study, we performed the full text screening of the these articles and identified 54 articles^14–66^ that contained quantitative measures of bone strength, bone mineral density (BMD) and composition (included parameters are presented in Table 1 and Supplementary Table 2). Twenty-six articles^42–66^ were excluded at this level with reasons described in Supplementary Table 3. Of note, 4 articles^42,55,58,59^ presented relevant bone measures in primates but were excluded due to insufficient quantity of any single measure of interest for quantitative synthesis. In the final meta-analysis, 28 articles^14–41^ were included, 20 regarding rats, and 8 regarding mice, flown on a total of 17 spaceflight missions, with a total of 60 rats and 60 mice being described (overview of included article is in Table 2).

**Table 1:** Included bone properties for meta-analysis.

Parameter	Description	Unit(s)
Measures of whole bone mechanical properties		
1. Maximum load	Load at which internal structure begins to fail^10^, peak load on the load-displacement curve	cm-dyne or N
2. Yield load	Load beyond which permanent deformation occurs^10^, the proportional limit on the load-displacement curve	N
3. Failure load	Load at which bone failure/fracture occurs.	N
4. Stiffness	Measure of whole bone resistance during elastic deformation^10^, slope of the initial linear portion of the load-displacement curve	dyne/rad or N/mm
5. Work to maximum load	Energy required to reach max load, area under the load-displacement curve until max load	Nmm or mJ
6. Work to failure load	Energy required to reach failure load, area under the load-displacement curve until failure load^69^	cm-dyne rad or Nmm
Measures of tissue-level mechanical properties		
1. Elastic modulus	Measure of tissue-level resistance to deformation, tissue-level stiffness^10^	GPa or MPa
2. Yield stress	Measure of stress at the yield point^94^	MPa
Bone density measure		
1. Bone mineral density	Mass of bone per unit volume or mass of bone per unit area	mg/mm^3^, g/cm^3^, mg/cm^3^, or mg/cm^2^
Bone composition measures		
1. Calcium content	Amount of calcium per mass of dry bone	g/100 g, mg/g, μg/mg, %dry weight, or mol/kg
2. Phosphorus content	Amount of phosphorus per mass of dry bone	g/100 g, mg/g, μg/mg, or mol/kg
3. Hydroxyproline content	Mass of hydroxyproline per mass of dry bone	mg/g or μg/mg
4. Osteocalcin content	Mass of osteocalcin per mass of dry bone	μg/mg, mg/g, or ng/mg

**Table 2:** Overview of articles included in meta-analysis.

Articles	Mission	Year	Days	Species	n_SF_	Type of control	Bone(s) analyzed	QS (/18)
Morey-Holton 1978a^14^	Cosmos 782	1975	19.5	Rats	4	GC, VC	Humerus	16^a^
Morey-Holton 1978b^15^	Cosmos 936	1977	18.5	Rats	4	GC, VC	Femur	14^a^
Prokhonchukov 1982^16^	Cosmos 1129	1979	18.5	Rats	5	GC, VC	Scapula	7
Rodgacheva 1984^17^					6		Femur	11
Patterson-Buckendahl 1985^18^	Spacelab 3	1985	7	Rats	6	GC	Humerus	10
Patterson-Buckendahl 1987^19^					6		Vertebrae (L3), Humerus	11
Shaw 1988^20^					6		Humerus, Tibia	11
Simmons 1986^21^					6		Vertebrae (T), Femur	11
Cann 1990^22^	Cosmos 1887	1987	12.5	Rats	5	GC, VC	Vertebrae (L4)	7.5^a^
Simmons 1990a^23^					6		Calvarium, Vertebrae (L5)	15
Vailas 1990a^24^					4		Humerus	17
Arnaud 1992^25^	Cosmos 2044	1989	14	Rats	5	GC, VC	Femur	13
Cann 1994^26^					5		Vertebrae (L5)	9^a^
Vailas 1992^27^					5		Humerus	16
Vailas 1994^28^					5		Vertebrae (L5)	8^a^
Lafage-Proust 1998^29^	STS-58	1993	14	Rats	5	GC	Parietal bone, Vertebrae (T), Humerus, Tibia	14
Chapes 1999^30^	STS-60	1994	8	Rats	6	GC, VC	Femur, Tibia	15
	STS-63	1995	8	Rats	6	GC, VC	Femur, Tibia	15
Bateman 1998^31^	STS-77	1996	10	Rats	6	VC	Humerus, Femur	14
Vajda 2001^32^	STS-78	1996	17	Rats	6	GC, VC	Femur	14
Zerath 2000^33^					6		Pelvic bone	14
Lloyd 2015^34^	STS-108	2001	12	Mice	12	GC	Vertebrae (L5), Femur, Tibia	14
Ortega 2013^35^	STS-118	2007	13	Mice	12	GC	Femur	14
Coulombe 2021^36^							Femur, Tibia	15
Zhang 2013^37^	STS-131	2010	15	Mice	7	GC	Calvariae	14
Gerbaix 2017^38^	Bion M1	2013	30	Mice	5	GC, VC	Vertebrae (T12 & L3), Femur	15
Gerbaix 2018^39^					5		Calcaneus, Navicular, Talus	15
Macaulay 2017^40^					6	GC	Calvariae	15
Coulombe 2021^36^	SpaceX-4	2014	21	Mice	10	GC	Femur, Tibia	14
Lee 2020^41^	SpaceX-19	2019	33	Mice	8	GC	Femur, Vertebrae (L2,3,5)	12

*Days* mission duration (days), *n*_*SF*_ sample size of spaceflight animal group. Control groups: *GC* ground control, *VC* vivarium control. For vertebrae region: *L* lumber, *T* thoracic, *C* caudal. *QS* quality score calculated according to Supplementary note 1.

^a^Indicates articles sourced from NASA Final Reports of Soviet missions.

### Overview of included bone parameters & control groups in the study

For each parameter, a minimum of 3 mission level outcomes were required to be included in this study. Mechanical properties included in meta-analysis consisted of 6 whole-bone mechanical properties: max load, yield load, failure load, stiffness, work to max load, and work to failure load (Supplementary Fig. 1a); and 2 tissue-level mechanical properties: elastic modulus and yield stress. All included measures of bone strength were from either torsional tests or 3-point bending tests (3PBT) conducted on long bones: tibia, femur, and humerus. For bone mineral density we included measurements from the following techniques: mercury porosimetry, dual-energy x-ray absorptiometry (DEXA), microcomputed tomography (μCT), peripheral quantitative computed tomography (pQCT) and calculated density obtained by authors by dividing the weight of cortical bone segment by its estimated volume. It is worth noting that 4 articles^37–40^ indicated that they measured tissue mineral density (TMD) rather than bone mineral density (BMD). However, we treated TMD and BMD identically, since the voxel size used in these μCT studies included contributions from porosity^67^. In addition, the polychromatic beam used in lab-based μCT leads to beam hardening effects, which further limits the accuracy of tissue mineralization measurements^68^. Bone composition data for 4 compounds present in bone, calcium, phosphorus, hydroxyproline, and osteocalcin, were included as the weight of the compound compared to the overall dry bone weight. The specific measurements present in each study are presented in Supplementary Table 4 and study characteristics used for covariate analysis in Supplementary Table 5.

For the purposes of analysis, two types of control animal groups were considered; a vivarium control group (VC) comprised of animals housed in standard laboratory habitats, and a ground control group (GC) where some or all aspects of spaceflight other than microgravity, including habitat, light/dark cycle, diet and forces of liftoff and re-entry were simulated. To assess the influence of microgravity, we calculated the normalized difference between SF and GC. To determine the possible effect of conditions associated with spaceflight other than microgravity on bone strength, we calculated the normalized difference between GC and VC.

### Heterogeneity, bias, and quality

Among the 13 included parameters, statistical heterogeneity was high (I^2^ > 75%) for 3 datasets; stiffness, yield stress, and bone density. Heterogeneity was moderate (55% > I^2^ > 40%) for 3 datasets: max load; work to max load; and elastic modulus. The remaining 7 datasets showed low (I^2^ < 25%) heterogeneity. The largest and most heterogeneous dataset, BMD, was used to assess global bias. From single study exclusion analysis, no single mission significantly affected global heterogeneity or outcome (Supplementary Fig. 1b). From cumulative study exclusion, 20% of studies were excluded prior to the dataset reaching homogeneity, and the outcome of the homogeneous dataset was similar to the complete dataset (Supplementary Fig. 1c). The funnel plot demonstrated uneven distribution; however, the presence of a specific bias was difficult to ascertain (Supplementary Fig. 1d). Regression analysis of article-level effect size as a function of quality score demonstrated that increased quality score was associated with decreased effect size magnitude for BMD and stiffness (Supplementary Fig. 1e, h). This association was however confounded by higher quality scores of newer articles, which also are describing mouse studies. Quality score was not associated with BMD article-level standard error (Supplementary Fig. 1f).

### Long bone mechanical properties

**Fig. 2:**
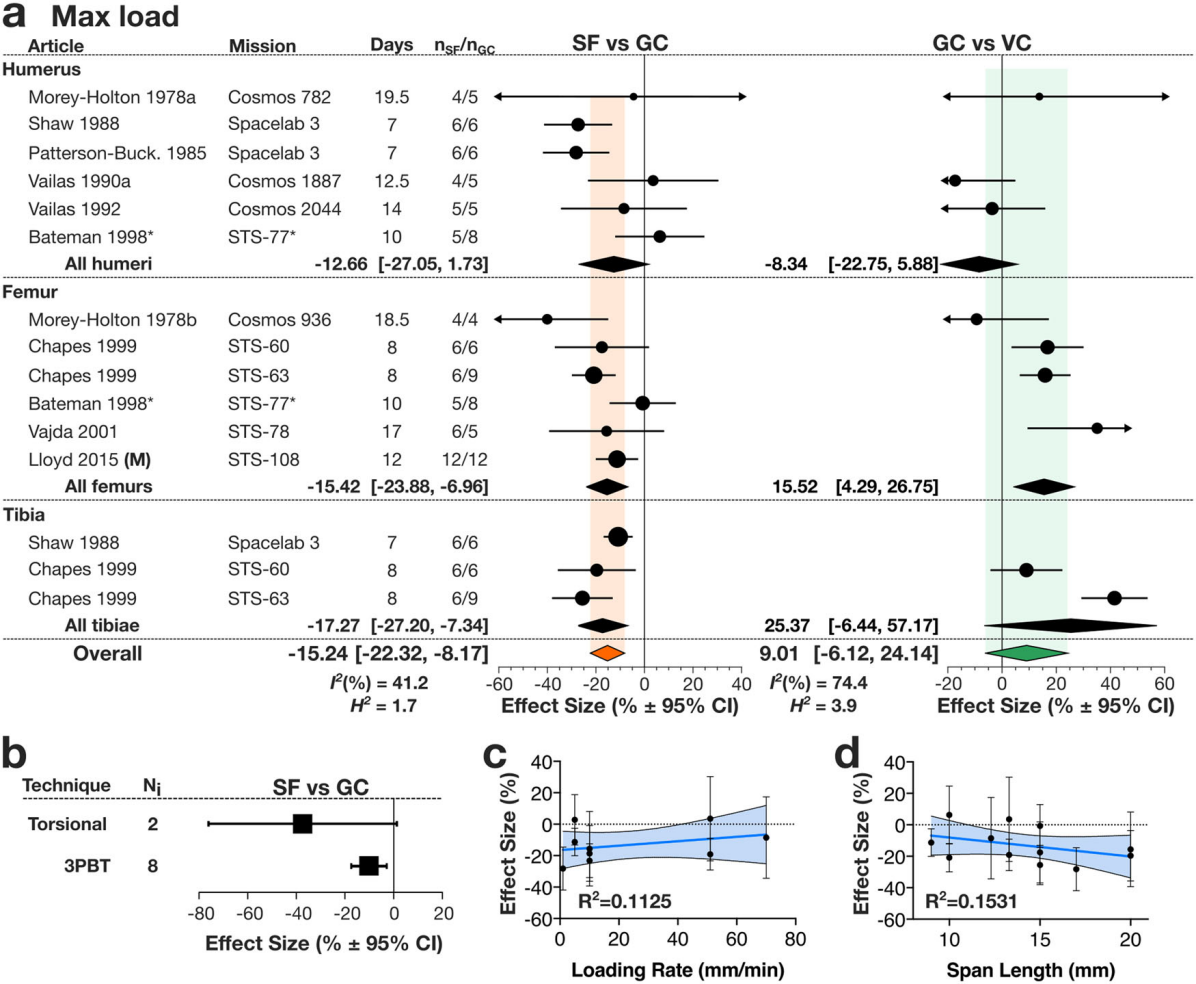
Spaceflight-induced changes in max load of bone in rodent. **a** Forest plot of changes in max load in humerus, femur and tibia in spaceflight animals (SF) compared to ground control (GC) (*Left*); and GC compared to vivarium control animals (VC) (*Right*). Missions are ordered by mission year; mission name, duration (Days), SF and GC sample sizes (n_SF_/n_GC_) are shown. Circle/line: effect size (%) and 95% CI, the size of the circle is proportional to the mission’s weight. *Black diamonds*: overall effect size and 95% CI for rats; *color diamonds*: overall effect size and 95% CI for rodents. *I*^2^ and *H*^2^ are for rodents. *indicates missions where there was no GC, and SF was compared to VC. **b** Subgroup analysis of measured max load by mechanical test: torsional test (Torsional) and 3-point bending tests (3PBT). Square/line: effect size (%) and 95% CI. N_i_: number of mission level outcomes. Meta-regression analysis of max load measures obtained by 3PBT as a function of loading rate (**c**) and span length (**d**) of the 3PBT machinery. Linear regression line (*dark blue*), its 95% CI (*light blue area*) and R^2^ are shown.

We first examined the effect of spaceflight on the bone strength parameters yield load, max load, and failure load obtained using 3-point bending (3PBT) or torsional tests conducted on long bones (Fig. 2). Spaceflight significantly reduced the max load in hindlimb long bones (Fig. 2a, Supplementary Table 6) with the calculated effect size representing the normalized difference in max load between SF and GC of −15.42% with a 95% CI of [−23.88, −6.96] in the femur, and –17.27% [−27.20, −7.34] in the tibia. The change in the forelimb long bones (humerus), was negative but not significant −12.66% [−27.05, 1.73]. For all the long bones, spaceflight significantly reduced the max load with the calculated effect size of −15.24% [−22.32,−8.17] (Fig. 2a*left*). In the femur was there a significant difference between GC and VC, with an increase of 15.52% [4.29, 26.75], however in other long bones and overall max load in GC and VC was not significantly different (Fig. 2a*right*). Subgroup analysis of effect of measurement technique on SF-induced changes to max load demonstrated no significant difference in outcomes resulting from torsional test and 3PBT (Fig. 2b). Among measures of max load derived from 3PBT machinery, neither loading rate nor span length of the supports were significantly associated with a change in outcome (Fig. 2c, d). Yield load and failure load decreased in SF compared to GC with a percent difference of −18.95% [−27.24, −10.66] and −10.41% [−21.99, 1.16] respectively, with only the change to yield load being statistically significant (Supplementary Tables 7 and 8). When yield load, max load, and failure load were normalized to weight of respective animal group at the time of sacrifice, we found the overall decrease in these parameters in SF animals compared to GC to be very similar: yield load −12.24% [−20.52, −3.95], max load −12.65% [−21.11, −4.18], failure load −11.36% [−21.47, −1.26], all of which were statistically significant (Supplementary Tables 9–11).

**Fig. 3:**
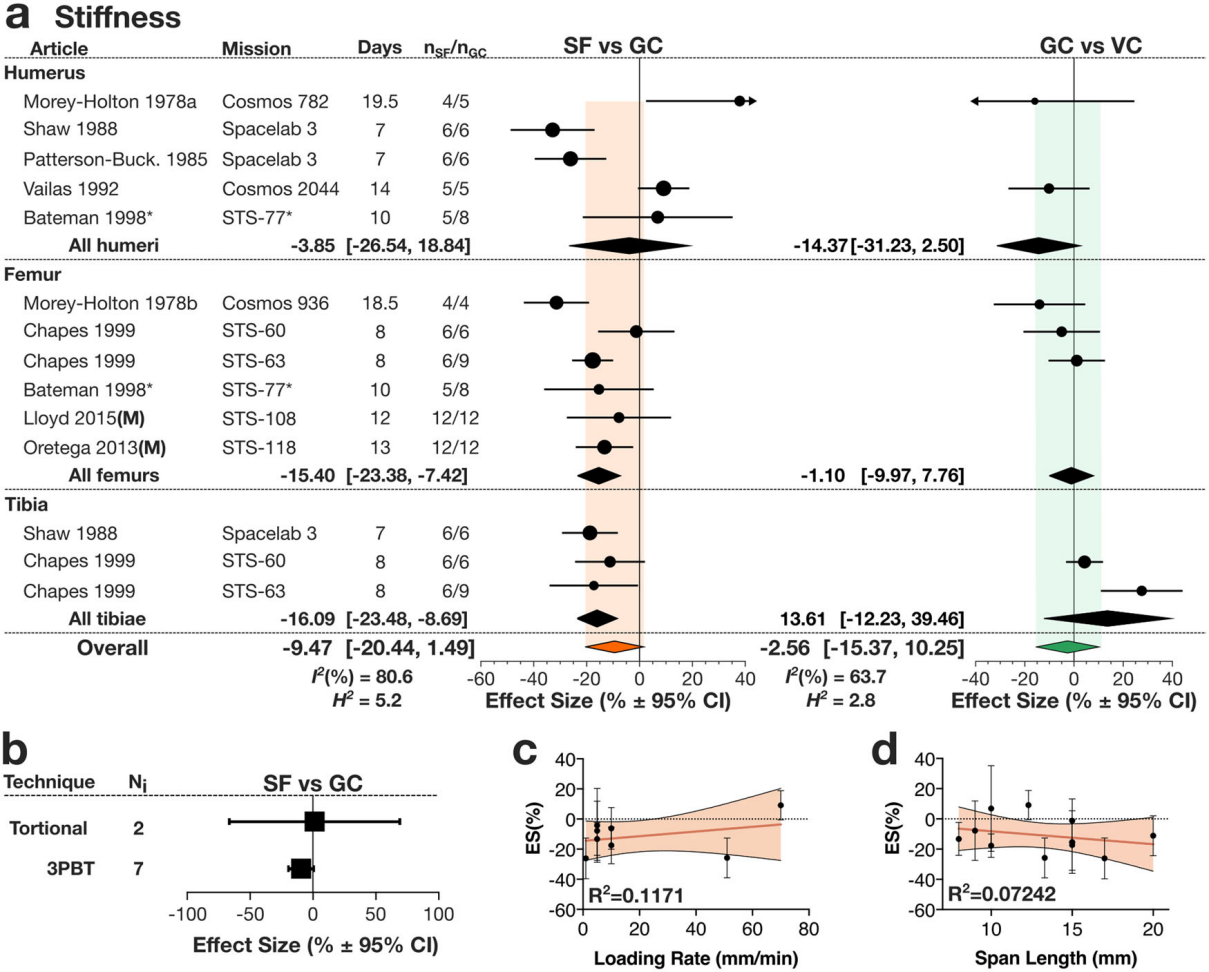
Spaceflight-induced changes in bone stiffness in rodents. **a** Forest plot of changes in stiffness in humerus, femur and tibia in spaceflight animals (SF) compared to ground control (GC) (*Left*); and GC compared to vivarium control animals (VC) (*Right*). Missions are ordered by mission year; mission name, duration (Days), SF and GC sample sizes (n_SF_/n_GC_) are shown. Circle/line: effect size (%) and 95% CI, the size of the circle is proportional to the mission’s weight. *Black diamonds*: overall effect size and 95% CI for rats; *color diamonds*: overall effect size and 95% CI for rodents. *I*^2^ and *H*^2^ are for rodents. *indicates missions where there was no GC, and SF was compared to VC. **b** Subgroup analysis of measured bone stiffness by mechanical test: torsional test (Torsional) and 3-point bending tests (3PBT). Square/line: effect size (%) and 95% CI. N_i:_ number of mission level outcomes. Meta-regression analysis of stiffness measures obtained by 3PBT as a function of loading rate (**c**) and span length (**d**) of the 3PBT machinery. Linear regression line (*dark blue*), its 95% CI (*light blue area*) and R^2^ are shown.

**Fig. 4:**
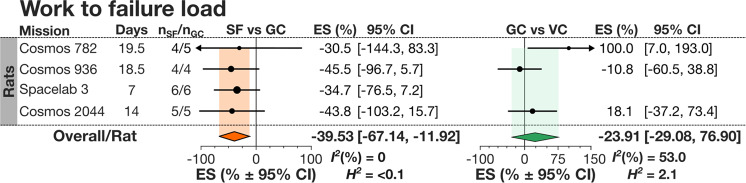
Spaceflight-induced changes in work to failure load in rats. Forest plot of changes in work to failure load in spaceflight animals (SF) compared to ground control (GC) (*Left*); and GC compared to vivarium control animals (VC) (*Right*). Missions are ordered by mission year; mission name, duration (Days), SF and GC sample sizes (n_SF_/n_GC_) are shown. Circle/line: effect size (%) and 95% CI, the size of the circle is proportional to the mission’s weight. *Orange diamonds*: overall effect size and 95% CI for rats.

Next, we assessed the effect of spaceflight on stiffness, work to max load, and work to failure load. Stiffness (Fig. 3, Supplementary Table 12) decreased in the hindlimbs of SF animals by −15.40% [−23.38, −7.42] in the femur and by −16.09% [−23.48, −8.69] in the tibia, while the change in humerus stiffness was not statistically significant, −3.85[−26.54, 18.84]. When all long bones were combined, the spaceflight-induced change to stiffness was not statistically significant (Fig. 3a*left*). There was no significant difference in long bones stiffness between GC and VC (Fig. 3a*right*). The effect of spaceflight on bone stiffness in long bones did not differ when sub-grouped by the measurement technique (Fig. 3b), and did not depend on loading rate (Fig. 3c) nor span length (Fig. 3d) in 3PBT. The data for work to max load and work to failure load were only available for rats. Both measures decreased in SF animals by −16.41% [−47.85, 15.03] and −39.53% [−67.14, −11.92] respectively, with the considerably larger and statistically significant decrease for work to failure load (Fig. 4, Supplementary Tables 13). Outcomes for stiffness, work to max load, and work to failure load were not significantly affected when normalized to weight at sacrifice (Supplementary Tables 14–16).

Given that included measures of bone strength were exclusively from torsional tests and 3PBT, reported tissue-level mechanical properties, elastic modulus and yield stress, were derived using engineering beam theory equations. In spaceflight rats, elastic modulus 1.64% [−19.98, 23.26] and yield stress 4.96 [−26.04, 35.97] exhibited no significant change from GC and had moderate to high heterogeneity (*I*^2^ ≥ 52%). Interestingly, elastic modulus demonstrated an overall significant difference between the ground control and vivarium control with the decrease in GC by −21.61% [−35.02, −8.19] compared to VC (Supplementary Tables 17 and 18). There were no available data reported for mice. The outcomes of elastic modulus and yield stress were unaffected when normalized to weight (Supplementary Tables 19 and 20).

### Bone Mineral Density

**Fig. 5:**
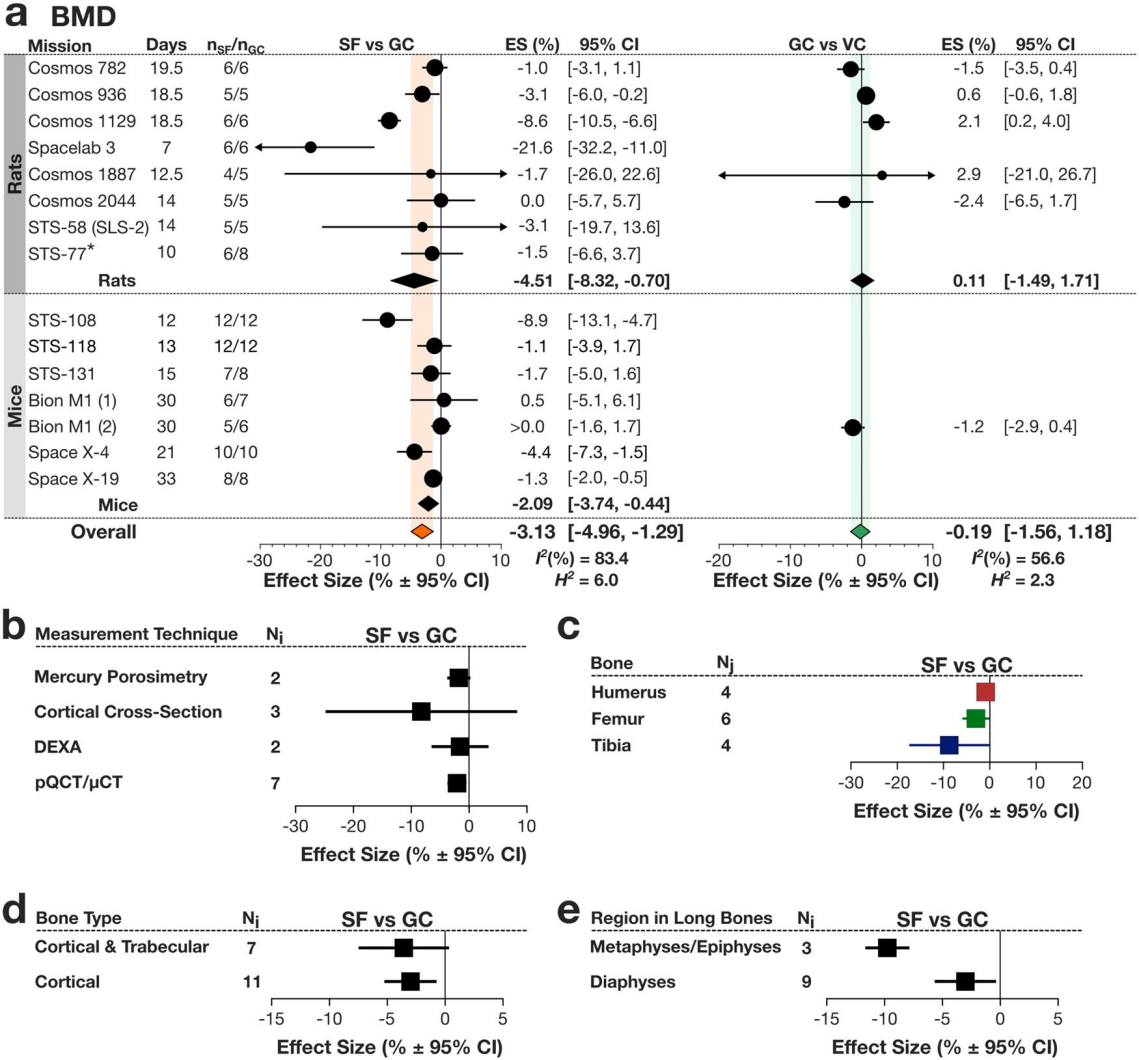
Spaceflight-induced changes in bone mineral density (BMD) in rodents. **a** Forest plot of changes in BMD in spaceflight animals (SF) compared to ground control (GC) (*Left*); and GC compared to vivarium control animals (VC) (*Right*). Missions are stratified by rodent species, and within each stratum, missions are ordered by mission year. Mission name, duration (Days), SF and GC sample sizes (n_SF_/n_GC_) are shown. Circle/line: effect size (%) and 95% CI, the size of the circle is proportional to the mission’s weight. *Black diamonds*: overall effect size and 95% CI for mice and rats; *color diamonds*: overall effect size and 95% CI for rodents. *I*^2^ and *H*^2^ are for rodents. **b** Subgroup analysis of changes in BMD by measurement technique, which included density derived from weight of cortical cross-section sample divided by volume determined either from mercury displacement (Mercury Porosimetry) or from geometric estimates (Cortical Cross-Sectional), as well as BMD obtained from DEXA, or pQCT/μCT**. c** Subgroup analysis of long bone BMD by the forelimb and hindlimb bones. **d** Subgroup analysis of all BMD outcomes by the bone type. **e** Subgroup analysis of long bone BMD by bone region. Square/line: effect size (%) and 95% CI. N_i_ number of mission level outcomes. N_j_ number of measurement level outcomes.

BMD was significantly lower in SF rats, −4.51% [−8.32, −0.70], and mice, −2.09 [−3.74, −0.44], compared to GC, with the overall effect for rodents of −3.13% [−4.96, −1.29] and high heterogeneity (*I*^*2*^ = 83.4%) (Fig. 5a*left*). GC and VC were not dfferent (Fig. 5a*right*). When stratified by the measurement technique, no significant difference was found between measurements obtained using mercury porosimetry, calculated from cross-sections, DEXA, and computed tomography (Fig. 5b). Spaceflight-induced decrease in BMD were statistically significant in the hindlimb bones, femur and tibia, but not the humerus of the forelimb (Fig. 5c). BMD measured from samples of bone that contained only cortical bone and samples that contained both cortical and trabecular bone demonstrated no significant difference in SF to GC outcomes (Fig. 5d). When only measures from long bones were considered, spaceflight-induced BMD deficits were greater in regions containing both cortical and trabecular bone (metaphyses and epiphyses) with a decrease of −9.8% [−11.7, −7.8] compared to regions containing only cortical bone (diaphyses) with a decrease of −3.0% [−5.7, −0.4] (Fig. 5e).

### Bone composition

**Fig. 6:**
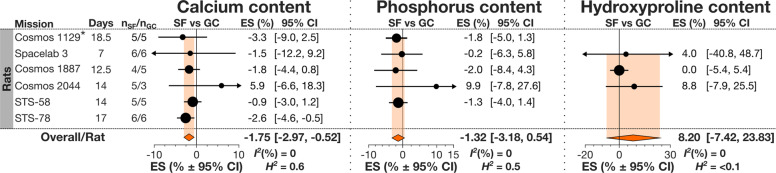
Spaceflight-induced changes to bone mineral composition in rats. Forest plot of changes in bone calcium (*left*), phosphorus (*middle*), and hydroxyproline (*right*) content of spaceflight animals (SF) compared to ground control (GC). Missions are ordered by mission year (old to recent). Mission name, duration (Days), SF and GC sample sizes (n_SF_/n_GC_) are shown. Circle/line: effect size (%) and 95% CI, the size of the circle is proportional to the mission’s weight. *Orange diamonds*: overall effect size and 95% CI for rats. *indicates missions where there was no GC, and SF was compared to VC.

The data for specific mineral and organic components of bone were only available for rats, and included homogeneous (*I*^*2*^ = 0%) datasets for bone calcium, phosphorus, hydroxyproline and osteocalcin. Spaceflight rats demonstrated a significant decrease in bone calcium content of −1.75% [−2.97, −0.52] (Fig. 6*left*). Phosphorus content in spaceflight rats demonstrated a similar but not significant decrease of −1.32% [−3.18, 0.54] (Fig. 6*middle*). Hydroxyproline content increased in the bone of spaceflight rats by 8.20% [−7.42, 23.83], although this change was not significant (Fig. 6*right*). GC to VC comparisons for bone composition parameters were not significantly different (Supplementary Tables 21–23). Osteocalcin content in bone was not affected by the spaceflight (Supplementary Table 24).

### Covariate analysis

**Fig. 7:**
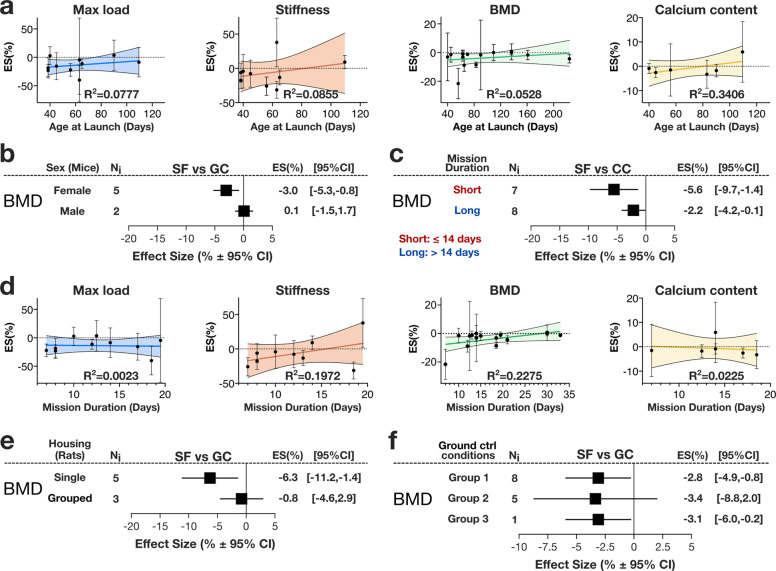
Covariate analysis of spaceflight-induced changes in bone strength and composition. **a** Meta-regression analysis of max load, stiffness, BMD, and calcium content as a function of age at launch of SF animals. Linear regression line (*dark color*), its 95% CI (*light color area*) and R^2^ are shown. Subgroup analysis of BMD by animal sex (**b**) and by short (14 days or less) and long (greater than 14 days) mission duration (**c**). Square/line: effect size (%) and 95% CI. N_i:_ number of mission level outcomes. **d** Meta-regression analysis of max load, stiffness, BMD, and calcium content as a function of mission duration. Linear regression line (*dark color*), its 95% CI (*light color area*) and R^2^ are shown. Subgroup analysis of BMD by single vs. grouped rat housing (**e**) and by how closely GC mimics SF conditions (**f**). For f: Group 1: GC housed in same habitat as the SF; Group 2: GC housed in same habitat as SF, the force of liftoff and/or re-entry were mimicked; Group 3: GC was mimicked by in-flight centrifuge. Square/line: effect size (%) and 95% CI. N_i:_ number of mission level outcomes.

We assessed the influence of covariates using subgroup and meta-regression analyses on the 4 parameters with 6 or more mission-level outcomes: max load, stiffness, BMD and calcium content. Animal related covariates included age at launch, age at sacrifice, strain, sex, source or dealer, weight of spaceflight animals at recovery or sacrifice and the difference in weight between the spaceflight and ground control animal groups (Δweight SF and GC). Linear regression analysis identified a weak association between age at launch and change in calcium content and no association with max load, stiffness, nor bone density (Fig. 7a). Similarly, only change in calcium content was weakly associated with animal age at sacrifice (Supplementary Fig. 2a). All spaceflight mice were of C57BL/6 strains, therefore subgroup analysis on animal strain was only applied to rats, in which the decreases to max load and stiffness were only significant in Sprague-Dawley rats, and not in Wistar rats, while density and calcium content changes were similar for both strains (Supplementary Fig. 2b). All spaceflight rats were male, therefore subgroup analysis for animal sex was only applied to mice. Comparing outcomes of BMD by sex in mice demonstrated a significant decrease in female but not in male mice, although the number of datasets for male mice was limited to 2 (Fig. 7b). Animals were obtained primarily from Institute of Experimental Endocrinology of Czeckolslovakia, Taconic Farms (Germantown, NY or affiliated facilities), or Jackson Laboratory (Bar Harbor, ME). Subgroup analysis of mission level outcomes by source of animal did not affect the outcomes (Supplementary Fig. 2c). Weight at time of sacrifice, or Δweight SF and GC did not significantly affected spaceflight outcomes (Supplementary Fig. 2d, e).

Mission-related covariates included mission duration, SF housing - either single or grouped, and year of mission launch. Subgroup analysis for short (<14 days) and long (≥14 days) duration mission demonstrated no significant difference between mission duration subgroups for any parameter (Fig. 7c, Supplementary Fig. 3a); however, the decrease in stiffness compared to GC was only significant in short durations missions, while the decrease in calcium was only significant in long-duration missions (Supplementary Fig. 3a). Longer mission duration was weakly associated with lower deficits in BMD in linear regression analysis (Fig. 7d). The max load, stiffness and BMD demonstrated greater deficits when rats were housed alone, although the difference between groups was not statistically significant (Fig. 7e, Supplementary Fig. 3b). There was no association between launch year and outcome (Supplementary Fig. 3c).

Study related covariates included sacrifice delay and the degree to which GC animals mimic the conditions of SF animals (GC condition). Sacrifice delay was weakly associated with decreased magnitude of max load, but did not affect other parameters (Supplementary Fig. 4a). BMD and calcium content outcomes were similar across all GC conditions (Fig. 7f, Supplementary Fig. 4b). While max load and stiffness appeared to be affected in some GC conditions, there were no consistent pattern (Supplementary Fig. 4b).

## Discussion

The objective of this study was to systematically review and quantitatively synthesize data regarding changes to bone strength and bone composition in rodents sent to space. We demonstrate that whole bone mechanical properties in spaceflight rodents were significantly decreased in their hindlimbs but not in the forelimbs. BMD was significantly decreased in spaceflight rodents. In spaceflight rats, bone calcium content was significantly lower, with a decrease in phosphorus and an increase in hydroxyproline that were not statistically significant. We were able to perform a limited analysis of the effect of some covariates on the SF-induced changes in bone strength and composition parameters. Spaceflight-induced deficits in BMD were significant in female mice, but not in male mice; decreases to bone strength parameters were only significant in Sprague-Dawley rats, and not in Wistar rats; bone strength and density were affected more in single-housed rats than group housed. However, the interactions between multiple confounding factors, for example age and sex, was not possible due to data paucity. Importantly, whole bone mechanical, BMD, and mineral composition properties were not significantly different between the ground control and vivarium animal groups, suggesting that microgravity is the primary factor causing these changes.

Our analysis only included measures of whole bone strength derived from 3-point bending tests or torsional tests. The relative change to bone strength from these two loading modes are considered to be comparable as they both depend on the underlying geometric and material properties^8^ of the tested region which is composed of cortical bone in long bone diaphysis. We found that yield load and max load were significantly lower in SF with estimated decreases of −18.95% [−27.24, −10.66] and −15.24% [−22.32,−8.17] respectively, while a decrease in bone stiffness of −9.47 [−20.44,1.49] was not significant. We found that work to failure load, which represents the area under the force-displacement curve until failure^69^, was the most affected parameter in spaceflight animals with a decrease of −39.53% [−67.14,−11.92], indicating a significant decrease to bone toughness, although toughness is also defined by fracture mechanics parameters^70^. Given that changes to stiffness, yield, max and failure load were all estimated to be less than half of the work to failure load magnitude of change, we can deduce that post-yield displacement (PYD), a measure of bone ductility^10^, may have been lower, potentially indicating increased bone brittleness in spaceflight animals. This is supported by two pieces of evidence. First, it has been reported that PYD has the greatest influence on work-to-fracture load^71^. Second, in two included studies, Patterson-Buckendahl et al.^18^ report of SpaceLab3 and Vailas et al.^27^ report of Cosmos 2044, max load and failure load occurred simultaneous. Tissue-level mechanical properties, elastic modulus and yield stress determined from engineering beam theory equations did not change in spaceflight animals. However, one must also consider the limitations of calculating tissue level properties from these equations, which has been reported to provide values that are greatly underestimated, with inconsistent and even inverse relative differences between experimental groups compared to the relative differences reported by nano-indentation measurements^72,73^. Therefore, our reported changes in tissue-level mechanical properties should be interpreted with caution. Thus, whole bone mechanical properties are significantly reduced in spacefaring rodents.

It has been reported that the whole bone mechanical properties depend on its mass, geometry and material compositional properties^8–10^. We demonstrated a significant decrease in BMD of cortical bone diaphysis: −3.0% [−5.7, −0.4]. Comparing the change in BMD to the changes in bone strength support the notion that changes to BMD alone may not explain the changes to bone strength^9,11^. We previously reported that in SF animals cortical bone area decreased significantly by −5.9% [−8.0, −3.8] and cortical thickness decreased by −4.7% [−13.7,4.4] while there was no significant change to marrow area^13^. Thus, cortical bone mass decreased during spaceflight with no increase in total cross-sectional area, which otherwise may have increased bone strength^10,70^. We also previously reported significant reductions in histomorphometric cortical bone formation indices only on the periosteal surface^13^. These SF-induced alterations in cortical microstructure due to imbalanced bone (re)modeling are consistent with the reduction of bone strength in SF animals.

Our study suggests that alterations in bone composition properties due to SF also contributed to the altered bone strength. In the current study, we have demonstrated that bone calcium content significantly decreased in SF rats compared to GC, with a trend of a decrease in phosphorus content, and a relative increase in the organic component of bone quantified by the increase in hydroxyproline, an amino acid unique to collagen is used as a relative measure of collagen content. There was no available data regarding calcium, phosphorus or hydroxyproline content in SF mice, and thus possible species differences could not be determined. Other factors including HA crystallinity^10,69,70^, presence of microcracks^74^, and changes in cortical bone porosity^11,70,75^ also may have significant effects on bone strength. Although the effect of spaceflight on HA crystallinity^38^, and cortical porosity^39^ were measured in Bion M1 mission, we lacked sufficient data of these outcome measures for meta-analysis. While many common methods used today to measure mineral and matrix properties such as quantitative backscattered electron imaging, nanoindentation, small angle x-ray scattering, Fourier transform infrared spectroscopy, and Raman spectroscopy^76^, they were not performed in enough studies to include in our analyses. Thus, our study identifies a significant gap in our knowledge of the degree to which bone tissue level properties are affected by microgravity.

Where it was possible, we investigated the effects of covariates on bone strength, density and composition outcomes. Similar to our previous findings^13^, neither mission duration, nor age at launch were associated with significant changes in measured parameter, likely due to the relatively short mission durations, up to 33 days, as well as the younger age of included animals. We confirmed that housing type had a significant effect on SF-induced changes. In rodents housed individually during spaceflight, a greater decrease in bone stiffness, max load, and BMD was observed compared to animals housed in groups. A hindlimb unloading study that directly compared the effect of unloading on single-housed mice and those housed in pairs demonstrated that several immune and hypothalamic-pituitary-adrenal axis responses were significantly different in these groups, suggestion strong contribution of social isolation to physiological responses to unloading^77^. However, in vivo mouse tibial loading studies performed on Earth have shown that the response to loading in male mice was reduced when mice were group housed, compared to individually housed mice, likely due to increased mechanical strains engendered in the tibiae during group-housed fighting activities that masked the bone (re)modeling response to loading^78^. We have also identified a potentially important difference between the responses to spaceflight in male and female mice, where only in female mice the spaceflight-induced deficits in BMD were significant. However, low number of studies with male mice and no studies with female rats presented a major limitation for further analysis.

We have found significant regional differences in the bone response to spaceflight. The change in BMD in the metaphyses of long bones was greater than the change in the diaphysis. This trend is consistent with our previous report examining bone architecture, where a greater reduction in trabecular bone compared to cortical bone was observed^13^. We have found that spaceflight-induced deficits in maximum load, stiffness and BMD were higher in the hindlimb bones compared to the forelimb bone, supporting a region dependent changes in bone health due to SF, which was similar to humans, for which the magnitude of bone loss was the highest in the legs, while arms were unaffected^6^. Previously, we reported a trend to higher trabecular bone deficits in distal skeletal regions compared to axial regions^13^. When we specifically analyzed the changes in humerus, femur and tibia, we found that spaceflight-induced changes in trabecular bone volume fraction (Tb.BV/TV) were −15.3% [−21.0, −9.7] in humerus, −29.0% [−33.5, −24.5] for femur and −24% [−30.5, −17.5] for tibia. This is also confirmed by in flight measurements of BMD using DEXA reported for SpaceX-19 mission, which reported that after 28 days of spaceflight decrease in BMD was observed in the femur and not the humerus^41^. Analysis of movement of mice sent to the International Space Station, noted forelimb ambulation during the first half of the mission as key in-flight activity^79^. These data suggests that the increased use of the forelimbs may help to preserve bone health in this region.

The limitations of this study included, *i*) variations in experimental designs between missions, *ii*) inconsistent reporting, *iii*) variations in measures of BMD, and *iv*) use of skeletally immature, growing animals. Limitations *i* and *ii* have been explored in detail in Fu & Goldsmith et al.^13^. In brief, mission designs and associated experiments have changed over time, and the included control group varied in terms of degree in which they mimic spaceflight-associated stressors. It was quite noticeable that reporting of certain parameters changes with time. For example, measures of whole bone mechanical properties were reported for all, but one spaceflight mission involving rats. In contrast, only 2 of the 6 included studies on spaceflight missions involving mice report whole bone mechanical properties. Similarly measures of bone calcium and phosphorus concentrations were only reported in rat missions, with no available data for mice. When grouping mission SF to GC outcomes by degree to which control group mimic spaceflight conditions, no clear association was observed suggesting that the microgravity is the main driver of the changes. Secondly, we observed that reported animal treatment was not consistent across publications. One example of this inconsistency is the great variation in reported sacrifice delays of SF animals among articles describing identical missions (Supplementary Table 5). The third set of limitations was related to the use of several different measurement technique to assess BMD. Among these techniques, some measures were more precise such as using μCT, others less so, such as estimating BMD by the weight of the bone sample, divided by the volume calculated as the cross-sectional area of the sample multiplied by its thickness. Four studies indicated that they report bone tissue mineral density^37–40^, however the smallest voxel size used was 9 μm, while a resolution of 1 μm is required to distinguish cortical vasculature microarchitecture^67^. For future studies, it would be valuable to also have analyses of bones using synchrotron-based tomography where smaller voxel sizes are possible and more accurate tissue mineral density can be determined without beam hardening artifacts that are present with lab-based computed tomography^68^. The final set of limitations was related to the use of skeletally immature rodents, particularly rats. Only one study included animals older than 6 months of age, and average age was ~ 11 weeks for rats, and 20 weeks for mice. C57BL/6 mice reach peak cancellous bone mass at 8–12 weeks of age. They achieve peak adult cortical bone density in the femur by 16 weeks^80^ and whole bone strength in bending and torsion peaks by 20 weeks of age^81^. Rats are skeletally mature at 6–9 months of age^82^. Since on average included mice were closer to skeletal maturity, this may explain why the decrease to BMD was less severe to mice compared to SF rats. However, one must keep in mind that age-related changes in BMD and mechanical properties are genetic strain and sex dependent in both mice^83^ and rats^84^. It is clear from loading studies in rodents that young animals have a much greater bone formation and resorptive response to mechanical loading^85,86^. It remains less clear how SF-induced bone (re)modeling changes are affected by age, but a recent study by Coulombe et al.^36^ showed that mature 32-week-old female mice exposed to microgravity experienced greater bone loss than young 9-week-old mice with net skeletal growth. However, aged mice similarly showed a diminished recovery upon re-ambulation compared to adult mice^82^. We were not able to perform extensive strain and sex analysis, because of limited information. Subgroup analysis of animal sex for BMD in mice demonstrated potential difference between the responses in male and female mice, however only 2 groups of male mice were included both from the same Bion M1 mission. Mechanical loading studies in mice have observed sex-related differences in cortical bone^87,88^, but not cancellous bone^89^. Genetic strain-specific differences in mechanoresponsive that have been reported between C57BL6, Balb/c, and C3H/HeJ mice^90–92^. Future studies are needed to carefully examine how genetic strain, age and sex affect the mechano-adaptive response to SF.

The two meta-analytic studies (Fu & Goldsmith et al.^13^ and the current study) quantitatively summarize previously reported changes to bone architecture, turnover, composition and mechanical properties in spacefaring rodents. We demonstrated significant deterioration in bone health, including decreased measures of bone architecture, strength and composition, and altered bone turnover. Our analysis is important in providing solid quantitative estimates of the effect sizes with measures of variance, and in identifying gaps and directions for informing future spaceflight experiments. In addition to the need for more inflight measurements of bone mass and architecture, standardizing measurement techniques, expanding the studies of animal sex, strain, age and spaceflight duration is critically important for obtaining a clear picture on how bone is changed in microgravity and how these changes can be prevented.

## Methods

This study was conducted in compliance with the Preferred Reporting Items for Systematic Reviews and Meta-Analysis (PRISMA) statement. For the PRISMA Checklist, refer to Supplementary Table 1.

### Search strategy, inclusion criteria and quality assessment

The systematic search strategy used in this study was identical to that used in Fu & Goldsmith et al. 2021^13^. In brief a search strategy using terms related to bone, space travel, and animals was constructed and used to execute a search Medline, Embase, PubMed, BIOSIS Previews, and Web of Science on November 2nd, 2017, with an updated search being performed on November 1st, 2019. An additional search of the NASA Technical Reporting Service (NTRS) and articles referenced in the compendium of animal and cell spaceflight experiments compiled by Ronca et al.^12^ was performed manually. No language restrictions were applied to considered articles. Title and abstract screening, performed independently by SDC & SFC for the primary search and by SVK for the update, selected articles describing any non-human vertebrate sent to space. Studies that described humans, invertebrates or Earth-based spaceflight simulations were excluded. Primary full text screening (conducted independently by SDC, SFC & MG for primary and MG for update) selected articles describing the effects of spaceflight on bone health of mice, rats and primate. We included in the meta-analysis studies that presented quantitative measures of strength, density and composition of bones of the axial and appendicular skeleton in mice and rats that were on normal diet, were not pregnant, and did not have surgery other than sham. Only studies that presented measures of bone strength resulting from three-point bending tests (3PBT) or torsional tests were included as the relative changes in outcomes obtained using these loading modes were suggested to be comparable^71^. Of studies reporting strength measures, only Zernicke et al.^54^ and Vailas et al.^28^ reported useable data derived from compression test machinery. Gerbaix et al.^38^ reported hardness and elastic modulus results using nanoindentation, which precluded meta-analysis for these measures. Papers included in meta-analysis were scored on an 18-point scale for reporting quality (Supplementary note 1). If the outcomes of two separate missions were reported in a single article, quality score (QS) was assessed for each mission independently.

### Data extraction

The following data was extracted by MG and verified by SVK for all studies included in meta-analysis: mission name and duration; animal species and sample size (*n*) of spaceflight, ground control, and vivarium control groups (when applicable); bone type and bone region being measured; measurement technique; and mean and median in the 13 bone parameters (Table 1); standard errors, standard deviations, and/or interquartile ranges; days when measurements were taken. If the type of dispersion measure was not given, we assumed it to be a standard error to ensure a conservative estimate. If a range of sample sizes was reported, the smallest value was extracted. The following mission characteristics were also extracted for covariate analysis: animal strain, age at launch and sacrifice, weight at sacrifice or recovery, sex, source or dealer of animals, year of mission, spaceflight group sacrifice delays, single vs grouped spaceflight habitat, and treatment conditions of ground control group. Mission characteristics were pooled from all applicable articles. If articles report differing values for apparently identical samples, the data from the article with the higher quality score was included. If articles report conflicting values for a single mission characteristic, the most frequently reported was included if possible, otherwise, the value from the article with a higher quality score was included. If only an interval of time was provided for age at launch the mean value was used, if only an interval of time was provided for spaceflight animal sacrifice delay, the higher value was used. All alternate terms used for included parameters are in Supplementary Table 2.

### Measurement-level outcomes

This study included relevant data of two control groups: the vivarium control (VC) consisting of animals housed in standard laboratory habitats, and the ground control (GC) which modeled some or all aspects of spaceflight except for microgravity. Animals sent to space and subjected to artificial gravity (AG)^15^ were considered GC. When possible, GC was used as a comparison group, in missions without GC, VC was used as the comparator for spaceflight (SF). For each bone measurement *j*, the mean SF value, *μ*_*SFj*_, and the mean comparison control (CC) value, *μ*_*CCj*_ with their associated standard errors *se*(*μ*_*j*_), or standard deviations sd(*μ*_*j*_) were recorded_._ In instances where sd(*μ*_*j*_) was recorded, it was converted to *se*(*μ*_*j*_) as $se\left( {\mu _j} \right) = {{{\mathrm{sd}}}}\left( {\mu _j} \right)/\sqrt n$, where *n* is *n*_*SF*_ for spaceflight and *n*_*CC*_ for the corresponding control. For median *P* and interquartile range $x_{upper}$− $x_{lower}$, *μ*_*j*_ was calculated as $\mu _j = (x_{upper} + P + x_{lower})/3$ with: $se\left( {\mu _j} \right) = x_{upper} - x_{lower}/\sqrt n \times 2.7.$ We calculated measurement-level effect size as the normalized percent difference, *θ*_*j*_, between *μ*_*SFj*_ and *μ*_*CCj*_ using Eq. (1).

1 $$\theta _j = \frac{{\mu _{SF_j} - \mu _{CC_j}}}{{\mu _{CC_j}}} \times 100{{{\mathrm{\% }}}}$$

The cumulative standard error in percentage, *se*(*θ*_*j*_), was calculated assuming the two groups were independent using Eq. (2).

2 $$se\left( {\theta _j} \right) = \sqrt {\left( {\frac{{se\left( {\mu _{SF_j}} \right)}}{{\mu _{SF_j}}}} \right)^2 + \left( {\frac{{se\left( {\mu _{CC_j}} \right)}}{{\mu _{CC_j}}}} \right)^2} \times 100{{{\mathrm{\% }}}}$$

### Mission-level outcomes

When measurement level outcomes of multiple unique *b* bones or bone regions were recorded for mission *i*, mission-level effect sizes *θ*_*i*_ and standard error *se*(*θ*_*i*_) were calculated as unweighted means by Eqs. (3), (4) respectively.

3 $$\theta _i = \frac{{\mathop {\sum } \theta _j}}{b}$$

4 $$se\left( {\theta _i} \right) = \frac{{\mathop {\sum } se\left( {\theta _j} \right)}}{b}$$

For a single mission, Bion M1, the data for two animal groups were reported separately^38,39^. As a result, these two animal groups were treated as independent missions.

### Meta-analytic model and global outcome

Considering that we combine data from two different rodent species aboard spaceflight missions with highly heterogeneous methodologies, a random effects (RE) model was selected. In accordance with the RE model, global effect size, $\hat \theta$, was calculated using mission-level outcomes *θ*_*i*_ and their associated weight *w*_*i*_ via Eq. (5),

5 $$\hat \theta = \frac{{\mathop {\sum }\nolimits_i^N \left( {\theta _i \times w_i} \right)}}{{\mathop {\sum }\nolimits_i w_i}}$$

where *N* is the number of combined mission-level outcomes. Equation (6) was used to calculate weight of mission-level outcomes *w*_*i*_ using mission-level standard error *se*(*θ*_*i*_) and the DerSimonian-Laird interstudy variance estimator *τ*^2^. *τ*^2^ was calculated using Eqs. (7), (8), and (9).

6 $$w_i = \frac{1}{{se\left( {\theta _i} \right)^2 + \tau ^2}}$$

7 $$\tau ^2 = \frac{{Q - (N - 1)}}{c}$$

8 $$Q = \mathop {\sum}\nolimits_i {se\left( {\theta _i} \right)^{ - 2} \times } \left( {\left( {\theta _i - \frac{{\mathop {\sum }\nolimits_i se\left( {\theta _i} \right)^{ - 2} \times \theta _i}}{{\mathop {\sum }\nolimits_i se\left( {\theta _i} \right)^{ - 2}}}} \right)^2} \right)$$

9 $$c = \mathop {\sum}\nolimits_i {se\left( {\theta _i} \right)^{ - 2}} - \frac{{\mathop {\sum }\nolimits_i \left( {se\left( {\theta _i} \right)^{ - 2}} \right)^2}}{{\mathop {\sum }\nolimits_i se\left( {\theta _i} \right)^{ - 2}}}$$

Standard error of global effect size was calculated using Eq. (10).

10 $$se\left( {\hat \theta } \right) = \frac{1}{{\sqrt {\mathop {\sum }\nolimits_i^N w_i} }}$$

95% confidence intervals (CI) was calculated as 95% CI$= \hat \theta \pm {{{\mathrm{z}}}}_{(1 - {\upalpha}/2)} \times se( {\hat \theta } ) = \hat \theta \pm 1.96 \times SE( {\hat \theta } )$. All the above analysis was repeated for GC to VC comparisons, replacing instances of SF and GC with GC and VC respectively.

### Heterogeneity and publication bias analysis

Heterogeneity of global outcomes were reported as *H*^2^ and *I*^2^ which uses Cochran’s Q (Eq. (8)) as: $H^2 = \frac{Q}{{N - 1}}$, and $I^2 = \frac{{H^2 - 1}}{{H^2}}$. To assess the contribution of individual missions to global outcome and heterogeneity, we performed single data exclusion analysis, wherein one at a time each mission-level outcome was sequentially removed and heterogeneity statistics recalculated. In cumulative data exclusion analysis mission-level outcomes were excluded sequentially starting with those that contributed the highest heterogeneity. A funnel plot showing the distribution of $se\left( {\theta _i} \right)$ to *θ*_*i*_ was used to assess reporting bias. Independent of their contribution to heterogeneity or potential bias, we included all the studies in the final analysis.

### Additional analysis

The following 17 characteristics were used for covariate analysis: flight duration, strain of rats, sex of mice, source or dealer of animals, age at launch & sacrifice, weight at sacrifice/recovery, change in weight between SF and CC group, launch year, SF sacrifice delay, single vs grouped housing condition, the degree to which GC group mimic the environmental conditions of SF (GC conditions), bone or bone region measured, measurement technique, span length & loading rate of 3PBT, and article quality score. Subgroup analysis was performed by combining mission-level outcomes and standard error within each category for categorical variables sex, strain, animal source, single vs grouped housing conditions, GC conditions, and measurement technique, as well as for short (<14 days) and long (≥14 days) duration missions. Subgroup analysis for measurement-level outcomes was used for bone type or bone region analysis. Meta-regression analysis was performed on mission level outcomes for continuous variables: flight duration, launch year, age at launch & sacrifice, weight at sacrifice or recovery, and change in weight between SF and CC group. Meta-regression analysis on measurement-level outcomes was performed for span length & loading rate in 3PBT. For quality score, missions reported in a single article were combined to create a paper-level score,*θ*_*p*_ and associated $se\left( {\theta _P} \right)$ using Eqs. (3) and (4), which were used in linear regression. Subgroup analysis and meta-regression analysis was only performed on parameters with 6 or more mission-level outcomes. We have also estimated the effect of body mass on the long bone mechanical properties, which was previously suggested to be significant^71^, by examining the effect of normalizing the reported means, *μ*_*j*_, and standard errors, $se\left( {\mu _j} \right)$, to the mean body mass *BM* of the corresponding animal group at the time of sacrifice.

### Outcome reporting

We report effect size as percentage difference *ES(%)* between SF and GC animals or GC and VC animals with lower and upper limits of 95% CI as: *ES(%) [lower CI, Upper CI]*.

### Software

Endnote X7 and Rayyan were used for reference management. WebPlot digitizer was in part used for data extraction. Microsoft Excel (version 16.44) was used for data management and initial calculations. METALAB, a custom software developed by N Mikolajewicz^93^ was used for global outcome and heterogeneity calculations. Figure preparation was accomplished using METALAB, Inkscape (version 1.0.1), and PRISM (version 9.0.0).

### Reporting summary

Further information on research design is available in the Nature Research Reporting Summary linked to this article.

## Supplementary information

Supplementary Information

Reporting summary checklist

## Data Availability

Raw data can be made available upon reasonable request to author Matthew Goldsmith (matthew.goldsmith2@mail.mcgill.com).

## Code availability

No custom code was used for the purposes of conducting this research paper. METALAB, a custom software for MatLab developed by Mikolajewicz^93^ is readily available.
