# Supplementary material for: Bone strength and composition in spacefaring rodents: systematic review and meta-analysis
Source: NPJ Microgravity. 2022 Apr 13;8:10. doi: 10.1038/s41526-022-00195-7 (PMC9008045; doi:10.1038/s41526-022-00195-7)
Supplement: Supplementary file 1 — Supplementary Information [file 41526_2022_195_MOESM1_ESM.pdf]

<u>Supplementary Information</u> for the manuscript "Bone strength and composition in spacefaring rodents: systematic review and meta-analysis" by Matthew Goldsmith, Sequoia D. Crooks, Sean F. Condon, Bettina M. Willie, Svetlana V. Komarova

<u>Supplementary notes</u>

Supplementary note 1. Quality score checklist for included articles [1-28]

(Total of 18)

1. Mission title & flight duration are clearly stated (1)
2. Clear indication of: (maximum of 4)
   sex (1), age (1), weight postflight (1), and sample size: n_{SF} (1) of spaceflight animals
3. Study contains the following control groups: (maximum 2 points)
   ground control group (1), vivarium control group (1)
4. Specify housing conditions of: (maximum 2 points)
   spaceflight group: group vs single housing (0.5) and specific habitat (0.5);
   ground control group: specific conditions in reference to spaceflight group (1)
5. Time of sacrifice/measurements for spaceflight group (1);
6. All data was presented in a table (1) or in a graph form (0);
7. When averaged data are presented clearly show sample size (*n*) for each measurement (1)
8. Clearly indicate all measurement units (including type of data spread) correctly (1)
9. Specific bone region from which measurements are taken is defined (1) and measurement techniques used are indicated (1)
10. Data regarding the following bone parameters shown: (maximum 3 points)
    Mechanical strength parameters (1), density parameters (1), mineral composition parameters (1)

Supplementary Tables

Supplementary Table 1. PRISMA Checklist

Section/topic	#	Checklist item	Reported on page #
**TITLE**			
Title	1	Identify the report as a systematic review, meta-analysis, or both.	1
**ABSTRACT**			
Structured summary	2	Provide a structured summary including, as applicable: background; objectives; data sources; study eligibility criteria, participants, and interventions; study appraisal and synthesis methods; results; limitations; conclusions and implications of key findings; systematic review registration number.	2
**INTRODUCTION**			
Rationale	3	Describe the rationale for the review in the context of what is already known.	3,4
Objectives	4	Provide an explicit statement of questions being addressed with reference to participants, interventions, comparisons, outcomes, and study design (PICOS).	4
**METHODS**			
Protocol and registration	5	Indicate if a review protocol exists, if and where it can be accessed (e.g., Web address), and, if available, provide registration information including registration number.	The study was not registered
Eligibility criteria	6	Specify study characteristics (e.g., PICOS, length of follow-up) and report characteristics (e.g., years considered, language, publication status) used as criteria for eligibility, giving rationale.	19,20
Information sources	7	Describe all information sources (e.g., databases with dates of coverage, contact with study authors to identify additional studies) in the search and date last searched.	19
Search	8	Present full electronic search strategy for at least one database, including any limits used, such that it could be repeated.	Refer to previous publication [29]
Study selection	9	State the process for selecting studies (i.e., screening, eligibility, included in systematic review, and, if applicable, included in the meta-analysis).	19,20, suppl note 1
Data collection process	10	Describe method of data extraction from reports (e.g., piloted forms, independently, in duplicate) and any processes for obtaining and confirming data from investigators.	20,21
Data items	11	List and define all variables for which data were sought (e.g., PICOS, funding sources) and any assumptions and simplifications made.	Table 1
Risk of bias in individual studies	12	Describe methods used for assessing risk of bias of individual studies (including specification of whether this was done at the study or outcome level), and how this information is to be used in any data synthesis.	23,24
Summary measures	13	State the principal summary measures (e.g., risk ratio, difference in means).	23,25

Section/topic	#	Checklist item	Reported on page #
Synthesis of results	14	Describe the methods of handling data and combining results of studies, if done, including measures of consistency (e.g., I^2) for each meta-analysis.	21-24
Risk of bias across studies	15	Specify any assessment of risk of bias that may affect the cumulative evidence (e.g., publication bias, selective reporting within studies).	23,24
Additional analyses	16	Describe methods of additional analyses (e.g., sensitivity or subgroup analyses, meta-regression), if done, indicating which were pre-specified.	24,25
**RESULTS**			
Study selection	17	Give numbers of studies screened, assessed for eligibility, and included in the review, with reasons for exclusions at each stage, ideally with a flow diagram.	4,5, Fig. 1, Suppl Table 3
Study characteristics	18	For each study, present characteristics for which data were extracted (e.g., study size, PICOS, follow-up period) and provide the citations.	5,6, Table 2, Suppl Tables 4, 5
Risk of bias within studies	19	Present data on risk of bias of each study and, if available, any outcome level assessment (see item 12).	6,7, Fig. 2, Suppl Fig. 1
Results of individual studies	20	For all outcomes considered (benefits or harms), present, for each study: (a) simple summary data for each intervention group (b) effect estimates and confidence intervals, ideally with a forest plot.	Fig. 3-7, Suppl Tables 6-23
Synthesis of results	21	Present results of each meta-analysis done, including confidence intervals and measures of consistency.	7-10, Fig. 3-6, Suppl Tables 6-23
Risk of bias across studies	22	Present results of any assessment of risk of bias across studies (see Item 15).	Fig. 2, Suppl Fig. 1
Additional analysis	23	Give results of additional analyses, if done (e.g., sensitivity or subgroup analyses, meta-regression [see Item 16]).	10,11, Fig. 2,7, Suppl Fig. 2-4
**DISCUSSION**			
Summary of evidence	24	Summarize the main findings including the strength of evidence for each main outcome; consider their relevance to key groups (e.g., healthcare providers, users, and policy makers).	11-16
Limitations	25	Discuss limitations at study and outcome level (e.g., risk of bias), and at review-level (e.g., incomplete retrieval of identified research, reporting bias).	16-18
Conclusions	26	Provide a general interpretation of the results in the context of other evidence, and implications for future research.	18,19
**FUNDING**			
Funding	27	Describe sources of funding for the systematic review and other support (e.g., supply of data); role of funders for the systematic review.	25,26

Supplementary Table 2. Alternative terms used for included parameters

Parameter	Alternate Term
**Whole bone mechanical properties**	
Max load	- Ultimate torque [1,2]<br>- Strength at max force [17]<br>- Max strength [18]<br>- Max force [21]
Yield load	- Load at proportional limit [11]<br>- Elastic strength [18]<br>- Elastic force [21]
Failure load	- Failure strength [18]<br>- Failure force [21]
Stiffness	N/A
Work to maximum load	- Energy to Max Load [7,11]
Work to failure	- Strain Energy [1,2]<br>- Energy to failure [7]
**Tissue-level mechanical properties of bone**	
Elastic Modulus	N/A
Yield Stress	- Tensile yield stress [7]<br>- Tensile stress at proportional limit [11]
**Bone density**	
Bone Mineral Density (BMD)	- Density [1,2]<br>- Cortical bone density [14]<br>- vBMD [21,23]<br>- TMD [24-27]
**Bone Composition Measures**	
Calcium content	N/A
Phosphorus content	N/A
Hydroxyproline content	N/A
Osteocalcin content	N/A

Supplementary Table 3. Excluded articles with quantitative measures of bone strength, density or composition

Article Reference	Mission	Species	Reason for Exclusion
Mack 1971[30]	Biosatellite III	Primate	Insufficient BMD data in primates for meta-analysis
Asling 1978 [31]	Cosmos 782	Rats	Report incompatible measure of mineral densities defined as "percent of non-porous structure"
Spengler 1979 [32]	Cosmos 936	Rats	Report neither raw data nor measure of variance
Spengler 1983 [33]	Cosmos 936	Rats	Data presented in Morey-Holton 1978b[2]
Kazarian 1981a [34]	Cosmos 1129	Rats	Bone mechanical properties derived from compression tests lacked measurement level variance useable for analysis
Kazarian 1981b [35]	Cosmos 1129	Rats	Both data and reason for exclusion identical to that of Kazarian 1981a[35]
Eurell 1983 [36]	Cosmos 1129	Rats	Report incompatible measure of calcium content
France 1983a [37]	Cosmos 1129	Rats	No true measure of variance
Russel 1985 [38]	Spacelab 3	Rats	Report nearly identical data to Simmons 1986[8] except measures of Hydroxyproline differed by a factor of 10^3
Arnaud 1990 [39]	Cosmos 1887	Rats	No true measure of variance reported, reported data identical to Mechanic 1990[40]
Mechanic 1990 [40]	Cosmos 1887	Rats	No true measure of variance reported, reported data identical to Arnaud 1990[39]
Simmons 1990b [41]	Cosmos 1887	Rats	Data presented in Simmons 1990a[10]
Vailas 1990b [42]	Cosmos 1887	Rats	Data presented in Vailas 1990a[11] & Simmons 1990a[10]
Zernicke 1990 [43]	Cosmos 1887	Rats	Bone mechanical properties derived from compression tests were excluded due to insufficient data for meta-analysis
Rakhmanov 1991 [44]	Cosmos 1887	Primates	Insufficient bone mineral content data in monkeys for meta-analysis
Arnaud 1994 [45]	Cosmos 2044	Rats	Data presented in Arnaud 1992[12]
Sinha 2002 [46]	STS-48	Rats	Report the included animals were neonatal when according to NASA technical reports, the rodents aboard STS-48 were 26 days old. Report number of vertebrae tested rather than number of animals. Lastly, the paper was published 11 years after the mission. Due to these reasons, we felt uncomfortable including it in meta-analysis.
Zerath 1996 [47]	Bion 10	Primates	Insufficient bone mineral content data in monkeys for meta-analysis
Arnaud 1997 [48]	Bion 10	Primates	Insufficient stiffness data in primates for meta-analysis
Yamada 1997 [49]	STS-58	Rats	No true measure of variance reported
Cavelina 1997 [50]	STS-62	Rats	Spaceflight rodents were ovariectomized (OVX)
Hatton 2002 [51]	STS-80	Rats	Both groups of spaceflight rodents had abnormal calcium diet
Bailey 2014 [52]	STS-131	Mice	Mechanical properties reported for vertebral disc, not bone.
Berg-Johansen 2016 [53]	Bion M1	Mice	Mechanical properties reported for vertebral disc, not bone. Measures of BMD specific to trabecular bone.
Chakraborty 2021[54]	SpaceX CRS-10	Mice	Measures of BMD from bones that were fractured prior to launch. Data from Sham operated mice was not presented in neither the main nor supplemental text.
Tominari 2019 [55]	SpaceX CRS-12	Mice	Measures of BMD specific to trabecular bone

Supplementary Table 4. Parameters included in meta-analysis

		Mechanical Properties									Density		Composition Properties				
**Mission**	**Articles**	**Bone**	**Max load**	**Yield load**	**Fail load**	**Stiff**	**Work to max load**	**Work to fail**	**EM**	**Yield stress**	**Bone**	**BMD**	**Bone**	**Ca**	**P**	**Hyp**	**OC**
Cosmos 782	Morey-Holton 1978a [1]	Humerus	✓			✓		✓			Humerus(D)	✓					
Cosmos 936	Morey-Holton 1978b [2]	Femur	✓			✓		✓			Femur(D)	✓					
Cosmos 1129	Prokhonchukov 1982 [3]												Scapula	✓	✓	✓	
	Rogdacheva 1984 [4]										Femur(D,E)	✓	Femur(E)	✓	✓		
Spacelab 3	Patterson-Buckendahl 1985 [5]	Humerus	✓		✓	✓							Vertebrae(L3)				✓
	Patterson-Buckendahl 1987 [6]												Vertebrae(L3), Humerus	✓	✓		✓
	Shaw 1988 [7]	Humerus, Tibia	✓	✓	✓	✓	✓	✓	✓	✓	Tibia(D)	✓					
	Simmons 1986 [8]												Vertebrae(T), Femur(D,M)	✓	✓	✓	
Cosmos 1887	Cann 1990 [9]												Vertebrae(L4)	✓	✓		✓
	Simmons 1990a [10]												Calvariae, Vertebrae(L5)	✓	✓	✓	
	Vailas 1990a [11]	Humerus	✓	✓			✓		✓	✓	Humerus(D)	✓	Humerus	✓	✓	✓	
Cosmos 2044	Arnaud 1992 [12]												Femur(D,M)	✓	✓	✓	✓
	Cann 1994 [13]												Vertebrae(L5)	✓			✓
	Vailas 1992 [14]	Humerus	✓		✓	✓	✓	✓	✓		Humerus(D)	✓	Humerus	✓		✓	
	Vailas 1994 [15]												Vertebrae(L5)			✓	
STS-58	Lafage-Proust 1998 [16]										Humerus, Femur, Tibia	✓	Parietal bone, Vertebrae(T), Tibia	✓	✓		
STS-60	Chapes 1999 [17]	Femur, Tibia	✓			✓											
STS-63		Femur, Tibia	✓			✓											
STS-77	Bateman 1998 [18]	Humerus,Femur	✓	✓	✓	✓					Femur, Tibia	✓					
STS-78	Vajda 2001 [19]	Femur	✓						✓	✓							
	Zerath 2000 [20]												Pelvic bone	✓			
STS-108	Lloyd 2015 [21]	Femur	✓	✓	✓	✓					Vertebrae(L5), Tibia(M)	✓					
STS-118	Ortega 2013 [22]	Femur				✓											
	Coulombe 2021 [23]										Femur, Tibia	✓					
STS-131	Zhang 2013 [24]										Calvariae	✓					
Bion M1	Gerbaix 2017 [25]										Femur(D)	✓					
	Gerbaix 2018 [26]										Calcaneus, Navicular, Talus	✓					
	Maucaulay 2017 [27]										Calvariae	✓					
SpaceX-4	Coulombe 2021 [23]										Femur, Tibia	✓					
SpaceX-19	Lee 2020 [28]										Femur(D)	✓					

Long bone sub-sections (epiphysis, E; metaphysis, M; diaphysis, D) are indicated. For vertebrae, the types (Lumbar, L; Thoracic, T) and number are indicated. Bone composition abbreviations: Ca = calcium, P = phosphorus, Hyp = hydroxyproline, OC = osteocalcin. Grey boxes indicate information not reported/not applicable.

Supplementary Table 5. Study characteristics for covariate analysis

<table>
<tr><th>Mission</th><th>Article (Reference)</th><th>Species</th><th>Strain</th><th>Source</th><th>Sex</th><th>Age (days) at launch</th><th>Age (days) at sacrifice</th><th>SF weight at sacrifice</th><th>ΔWeight SF – GC</th><th colspan="2">SF sacrifice delay (hours)</th><th>Group housed</th><th>GC conditions</th></tr>
<tr><td>Cosmos 782</td><td>Morey-Holton 1978a [1]</td><td>Rats</td><td>W</td><td>Czeck.</td><td>M</td><td>63</td><td>82.5</td><td>258g</td><td>-11g</td><td colspan="2">NR</td><td></td><td>2</td></tr>
<tr><td>Cosmos 936</td><td>Morey-Holton 1978b [2]</td><td>Rats</td><td>W</td><td>Czeck</td><td>M</td><td>63</td><td>81.5</td><td>304g</td><td>40g</td><td colspan="2">NR</td><td></td><td>3</td></tr>
<tr><td rowspan="2">Cosmos 1129</td><td>Prokhonchukov 1982 [3]</td><td rowspan="2">Rats</td><td rowspan="2">W</td><td rowspan="2">Czeck [34]</td><td rowspan="2">M</td><td rowspan="2">83 [34]</td><td rowspan="2">101.5 [34]</td><td rowspan="2">336g</td><td rowspan="2">-8g</td><td colspan="2" rowspan="2">NR</td><td rowspan="2"></td><td rowspan="2">2</td></tr>
<tr><td>Rogdacheva 1984 [4]</td></tr>
<tr><td rowspan="4">Spacelab 3</td><td>Patterson-Buckendahl 1985 [5]</td><td rowspan="4">Rats</td><td rowspan="4">SD</td><td rowspan="4">Taconic</td><td rowspan="4">M</td><td rowspan="4">56</td><td rowspan="4">64</td><td rowspan="4">248g</td><td rowspan="4">-10g</td><td>12</td><td rowspan="4">12</td><td rowspan="4"></td><td rowspan="4">1</td></tr>
<tr><td>Patterson-Buckendahl 1987 [6]</td><td>12</td></tr>
<tr><td>Shaw 1988 [7]</td><td>11</td></tr>
<tr><td>Simmons 1986 [8]</td><td>12</td></tr>
<tr><td rowspan="3">Cosmos 1887</td><td>Cann 1990 [9]</td><td rowspan="3">Rats</td><td rowspan="3">W</td><td rowspan="3">Czeck.</td><td rowspan="3">M</td><td rowspan="3">90</td><td rowspan="3">102.5</td><td rowspan="3">303g</td><td rowspan="3">-46g</td><td>NR</td><td rowspan="3">53</td><td rowspan="3">✓</td><td rowspan="3">2</td></tr>
<tr><td>Simmons 1990a [10]</td><td>56</td></tr>
<tr><td>Vailas 1990a [11]</td><td>53</td></tr>
<tr><td rowspan="4">Cosmos 2044</td><td>Arnaud 1992 [12]</td><td rowspan="4">Rats</td><td rowspan="4">W</td><td rowspan="4">Czeck.</td><td rowspan="4">M</td><td rowspan="4">109.5</td><td rowspan="4">123.5</td><td rowspan="4">338g</td><td rowspan="4">-5g</td><td>NR</td><td rowspan="4">11</td><td rowspan="4">✓</td><td rowspan="4">2</td></tr>
<tr><td>Cann 1994 [13]</td><td>NR</td></tr>
<tr><td>Vailas 1992 [14]</td><td>NR</td></tr>
<tr><td>Vailas 1994 [15]</td><td>11</td></tr>
<tr><td>STS-58</td><td>Lafage-Proust 1998 [16]</td><td>Rats</td><td>SD</td><td>Taconic</td><td>M</td><td>56</td><td>70</td><td>315g</td><td>-2g</td><td colspan="2">3</td><td></td><td>2</td></tr>
<tr><td>STS-60</td><td rowspan="2">Chapes 1999 [17]</td><td>Rats</td><td>SD</td><td>Charles River</td><td>M</td><td>38.5</td><td>46.5</td><td>255.9g</td><td>-10.8g</td><td colspan="2">6</td><td>✓</td><td>1</td></tr>
<tr><td>STS-63</td><td>Rats</td><td>SD</td><td>Taconic</td><td>M</td><td>38.5</td><td>46.5</td><td>270.4g</td><td>-30.2g</td><td colspan="2">4</td><td>✓</td><td>1</td></tr>
<tr><td>STS-77</td><td>Bateman 1998 [18]</td><td>Rats</td><td>SD</td><td>Taconic</td><td>M</td><td>40</td><td>50</td><td>252g</td><td>38g</td><td colspan="2">3</td><td>✓</td><td>N/A</td></tr>
<tr><td rowspan="2">STS-78</td><td>Vajda 2001 [19]</td><td rowspan="2">Rats</td><td rowspan="2">SD</td><td rowspan="2">Taconic</td><td rowspan="2">M</td><td rowspan="2">45</td><td rowspan="2">62</td><td rowspan="2">281.2g</td><td rowspan="2">11g</td><td>7</td><td rowspan="2">6</td><td rowspan="2">✓</td><td rowspan="2">1</td></tr>
<tr><td>Zerath 2000 [20]</td><td>6</td></tr>
<tr><td>STS-108</td><td>Lloyd 2015 [21]</td><td>Mice</td><td>C57BL/6J</td><td>Jackson Lab.</td><td>F</td><td>65</td><td>77</td><td></td><td></td><td colspan="2">3.5</td><td>✓</td><td>1</td></tr>
<tr><td rowspan="2">STS-118</td><td>Ortega 2013 [22]</td><td rowspan="2">Mice</td><td>C57BL/6</td><td rowspan="2">Charles River</td><td rowspan="2">F</td><td rowspan="2">63</td><td rowspan="2">76</td><td></td><td></td><td colspan="2" rowspan="2">6</td><td rowspan="2">✓</td><td rowspan="2">1</td></tr>
<tr><td>Coulombe 2021 [23]</td><td>C57BL/6N</td><td></td><td></td></tr>
<tr><td>STS-131</td><td>Zhang 2013 [24]</td><td>Mice</td><td>C57BL/C</td><td>Jackson Lab.</td><td>F</td><td>161</td><td>176</td><td></td><td></td><td colspan="2">4</td><td>✓</td><td>1</td></tr>
<tr><td rowspan="3">Bion M1</td><td>Gerbaix 2017 [25]</td><td rowspan="3">Mice</td><td>C57BL/6N</td><td rowspan="3">Shem. & Ov.</td><td rowspan="3">M</td><td rowspan="3">136.5</td><td rowspan="3">166.5</td><td></td><td></td><td>25</td><td rowspan="2">24</td><td rowspan="3">✓</td><td rowspan="3">1</td></tr>
<tr><td>Gerbaix 2018 [26]</td><td>C57BL/6</td><td></td><td></td><td>24</td></tr>
<tr><td>Maucaulay 2017 [27]</td><td>C57BL/6N</td><td></td><td></td><td colspan="2">15</td></tr>
<tr><td>SpaceX-4</td><td>Coulombe 2021 [23]</td><td>Mice</td><td>C57BL/6N</td><td>Taconic</td><td>F</td><td>224</td><td>245</td><td></td><td></td><td colspan="2">NR</td><td>✓</td><td>1</td></tr>
<tr><td>SpaceX-19</td><td>Lee 2020 [28]</td><td>Mice</td><td>C57BL/6N</td><td>Jackson Lab.</td><td>F</td><td>70</td><td>103</td><td></td><td></td><td colspan="2">8</td><td>✓</td><td>1</td></tr>
</table>

List of abbreviations are as follows. Animal strain: **W** = wistar rats, **SD** = Sprague-Dawley rats. Animal source: **Czeck** = Institute of experimental Endochronology, Czeckolslovakia, **Taconic** = Taconic Farms, Germantown, NY, or affiliated facilities, **Charles River** = Charles River Laboratories, Wilmington MA, **Jackson Lab.** = Jackson Laboratory; Bar Harbor, ME, **Shem. & Ov.** = Animal Breeding Facility branch of Shemyakin & Ovchinnikov Institute of Bioorganic Chemistry, Moscow, Russia. Sex: **M** = male, **F** = female. **NR** = not recorded, **N/A** = not applicable. GC conditions indicate the degree from 1 (poorest) to 3 (best) spaceflight associated conditions other than microgravity are mimicked in treatment of GC animals.

Supplementary Table 6. Max load

Bone	Article	Mission	Days	n_{SF}/n_{GC}	SF vs GC ES (%)	SF vs GC 95% CI	GC vs VC ES (%)	GC vs VC 95% CI
Humerus	Morey-Holton1978a	Cosmos 782	19.5	4/5	-4.5	[-78.3,69.2]	13.8	[-50.3, 77.9]
	Shaw1988	Spacelab 3	7	6/6	-27.4	[-41.4, -13.3]	NA	NA
	Patterson-Buckendahl1985	Spacelab 3	7	6/6	-28.2	[-41.9, -14.5]	NA	NA
	Vailas1990a	Cosmos 1887	12.5	4/5	3.5	[-23.3, 30.3]	-17.3	[-39.4, 4.9]
	Vailas1992	Cosmos 2044	14	5/5	-8.5	[-34.4, 17.4]	-3.6	[-23.2, 16.0]
	Bateman1998	STS-77*	10	5/8	6.3	[-12.1, 24.6]	NA	NA
**All humeri**					**-12.66**	**[-27.05, 1.73]**	**-8.43**	**[-22.75, 5.88]**
Femur	Morey Holton1978b	Cosmos 936	18.5	4/4	-40.1	[-65.2, -14.9]	-9.3	[-35.9, 17.3]
	Chapes1999	STS-60	8	6/6	-17.5	[-36.9, 1.9]	16.8	[3.5, 30.1]
	Chapes1999	STS-63	8	6/9	-20.9	[-29.9, -11.9]	15.9	[6.6, 25.3]
	Bateman1998	STS-77*	10	5/8	-0.7	[-14.4, 12.9]	NA	NA
	Vajda 2001	STS-78	17	6/5	-15.6	[-39.3, 8.1]	35.1	[9.4, 60.8]
	Lloyd 2015	STS-108	12	12/12	-11.3	[-20.0, -2.6]	NA	NA
**All femurs**					**-15.42**	**[-23.88, -6.96]**	**15.52**	**[4.29, 26.75]**
Tibia	Shaw1988	Spacelab 3	7	6/6	-10.8	[-16.8, -4.9]	NA	NA
	Chapes1999	STS-60	8	6/6	-19.7	[-35.7, -3.6]	9.0	[-4.2, 22.3]
	Chapes1999	STS-63	8	6/9	-25.6	[-38.0, -13.1]	41.5	[29.3, 53.7]
**All tibiae**					**-17.27**	**[-27.20, -7.34]**	**25.37**	**[-6.44, 57.17]**
**Overall**					**-15.24**	**[-22.32, -8.17]**	**9.01**	**[-6.12, 24.14]**
					I^2 **= 41.2**		I^2 **= 74.4**	

Supplementary Table 7. Yield load

Species	Flight	Days	n_{SF}	SF vs GC ES (%)	SF vs GC 95% CI
Rats	Spacelab 3	7	6	-16.0	[-34.4, 2.3]
	Cosmos 1887	12.5	4	-9.3	[-41.1, 22.5]
	STS-77*	10	5	2.3	[-32.5, 37.0]
**Rats Overall**				**-11.46**	**[-25.92, 3.00]**
Mice	STS-108	12	12	-22.6	[-32.7, -12.5]
**Rodents Overall**				**-18.95**	**[-27.24, -10.66]**
				I^2 **= 0**	

Supplementary Table 8. Failure load

				SF vs GC	
**Species**	**Flight**	**Days**	**n_{SF}**	**ES (%)**	**95% CI**
Rats	Spacelab 3	7	6	-22.3	[-38.4, -6.2]
	Cosmos 2044	14	5	-8.5	[-34.4, 17.4]
	STS-77*	10	5	2.8	[-17.3, 22.8]
**Rats Overall**				**-10.44**	**[-26.40, 5.51]**
Mice	STS-108	12	12	-7.9	[-30.6, 14.7]
**Rodents Overall**				**-10.41**	**[-21.99, 1.16]**
				**I^2 = 0**	

Supplementary Table 9. Normalized max load

				SF vs GC	
**Species**	**Flight**	**Days**	**n_{SF}**	**ES (%)**	**95% CI**
Rats	Cosmos 782	19.5	4	-0.5	[-74.3, 73.3]
	Cosmos 936	18.5	4	-48.0	[-73.1, -22.8]
	Spacelab 3	7	6	-19.0	[-30.2, -7.8]
	Cosmos 1887	12.5	4	19.2	[-7.6, 46.0]
	Cosmos 2044	14	5	-7.2	[-33.1, 18.7]
	STS-60	8	6	-15.2	[-32.9, 2.6]
	STS-63	8	6	-14.6	[-25.4, -3.9]
	STS-77*	10	5	-12.7	[-28.7, 3.3]
	STS-108	17	6	-18.9	[-42.6, 4.8]
**Rats Overall**				**-15.09**	**[-23.57, -6.62]**
Mice	STS-108	12	12	-1.1	[-9.8, 7.6]
**Rodents Overall**				**-12.65**	**[-21.11, -4.18]**
				**I^2 = 57.8**	

Supplementary Table 10. Normalized yield load

				SF vs GC	
**Species**	**Flight**	**Days**	**n_{SF}**	**ES (%)**	**95% CI**
Rats	Spacelab 3	7	6	-12.6	[-31.0, 5.7]
	Cosmos 1887	12.5	4	4.5	[-27.3, 36.3]
	STS-77*	10	5	-13.2	[-47.9, 21.6]
**Rats Overall**				**-9.19**	**[-23.64, 5.27]**
Mice	STS-108	12	12	-13.7	[-23.8, -3.6]
**Rodents Overall**				**-12.24**	**[-20.52, -3.95]**
				**I^2 = 0**	

Supplementary Table 11. Normalized failure load

Species	Flight	Days	n_{SF}	SF vs GC ES (%)	SF vs GC 95% CI
Rats	Spacelab 3	7	6	-19.2	[-35.2, -3.1]
	Cosmos 2044	14	5	-7.2	[-33.1, 18.7]
	STS-77*	10	5	-12.7	[-32.8, 7.3]
**Rats Overall**				**-14.84**	**[-26.14, -3.55]**
Mice	STS-108	12	12	2.6	[-20.0, 25.3]
**Rodents Overall**				**-11.36**	**[-21.47, -1.26]**
				$I^2 = 0$	

Supplementary Table 12. Stiffness

Bone	Article	Flight	Days	n_{SF}/n_{GC}	SF vs GC ES (%)	SF vs GC 95% CI	GC vs VC ES (%)	GC vs VC 95% CI
Humerus	Morey-Holton1978a	Cosmos 782	19.5	4/5	37.9	[2.5, 73.3]	-15.9	[-56.4, 24.5]
	Shaw1988	Spacelab 3	7	6/6	-32.9	[-48.7, -17.0]	*NA*	*NA*
	Patterson-Buckendahl1985	Spacelab 3	7	6/6	-26.1	[-39.6, -12.6]	*NA*	*NA*
	Vailas1992	Cosmos 2044	14	5/5	9.1	[-0.6, 18.8]	-14.0	[-32.6, 4.5]
	Bateman1998	STS-77*	10	5/8	6.9	[-21.4, 35.2]	*NA*	*NA*
**All humeri**					**-3.85**	**[-26.54, 18.84]**	**-14.37**	**[-31.23, 2.50]**
Femur	Morey-Holton1978b	Cosmos 936	18.5	4/4	-31.4	[-43.7, -19.1]	-10.2	[-26.6, 6.3]
	Chapes1999	STS-60	8	6/6	-1.2	[-15.6, 13.2]	-5.0	[-20.6, 10.5]
	Chapes1999	STS-63	8	6/9	-17.7	[-25.5, -10.0]	4.3	[-3.2, 11.8]
	Bateman1998	STS-77*	10	5/8	-15.3	[-36.0, 5.3]	*NA*	*NA*
	Lloyd2015	STS-108	12	12/12	-7.8	[-27.5, 11.9]	*NA*	*NA*
	Oretega2013	STS-118	13	12/12	-13.3	[-24.1, -2.4]	*NA*	*NA*
**All femurs**					**-15.40**	**[-23.38, -7.42]**	**-1.10**	**[-9.97, 7.76]**
Tibia	Shaw1988	Spacelab 3	7	6/6	-18.8	[-29.3, -8.2]	*NA*	*NA*
	Chapes1999	STS-60	8	6/6	-11.2	[-24.4, 2.1]	1.1	[-10.4, 12.6]
	Chapes1999	STS-63	8	6/9	-17.3	[-34.0, -0.5]	27.5	[10.9, 44.1]
**All tibiae**					**-16.09**	**[-23.48, -8.69]**	**13.61**	**[-12.23, 39.46]**
**Overall**					**-9.47**	**[-20.44, 1.49]**	**-2.56**	**[-15.37, 10.25]**
					$I^2 = 80.6$		$I^2 = 63.7$	

Supplementary Table 13. Work to max load

Species	Flight	Days	n_{SF}/n_{GC}	SF vs GC ES (%)	SF vs GC 95% CI	GC vs VC ES (%)	GC vs VC 95% CI
Rats	Spacelab 3	7	6	-27.6	[-59.0, 3.9]	*NA*	*NA*
	Cosmos 1887	12.5	4/5	12.7	[25.6, 51.0]	-16.0	[-48.0, 16.0]
	Cosmos 2044	14	5	-43.8	[-103.2, 15.7]	18.1	[-37.2, 73.4]
**Rats Overall**				**-16.41**	**[-47.85, 15.03]**	**-6.69**	**[-36.47, 23.09]**
				$I^2 = 43.1$		$I^2 = 8.7$	

Supplementary Table 14. Normalized stiffness

				SF vs GC	
**Species**	**Flight**	**Days**	**n_{SF}**	**ES (%)**	**95% CI**
Rats	Cosmos 782	19.5	4	43.8	[8.4, 79.2]
	Cosmos 936	18.5	4	-40.4	[-52.8, -28.1]
	Spacelab 3	7	6	-22.9	[-36.2, -9.6]
	Cosmos 2044	14	5	10.7	[1.0, 20.4]
	STS-60	8	6	-2.2	[-16.1, 11.6]
	STS-63	8	6	-8.3	[-20.5, 4.0]
	STS-77*	10	5	-18.7	[-43.2, 5.8]
**Rats Overall**				**-7.84**	**[-24.50, 8.82]**
Mice	STS-108	12	12	2.8	[-16.9, 22.5]
	STS-118	13	12	1.6	[-9.3, 12.4]
**Mice Overall**				**1.86**	**[-7.65, 11.37]**
**Rodents Overall**				**-5.84**	**[-18.89, 7.20]**
				**I^2 = 86.5**	

Supplementary Table 15. Normalized work to max load

				SF vs GC	
**Species**	**Flight**	**Days**	**n_{SF}**	**ES (%)**	**95% CI**
Rats	Spacelab 3	7	6	-24.6	[-56.1, 6.8]
	Cosmos 1887	12.5	4	29.8	[-8.5, 68.1]
	Cosmos 2044	14	5	-42.9	[-102.4, 16.5]
**Rats Overall**				**-9.88**	**[-51.93, 32.17]**
				**I^2 = 67.5**	

Supplementary Table 16. Normalized work to failure

				SF vs GC	
**Species**	**Flight**	**Days**	**n_{SF}**	**ES (%)**	**95% CI**
Rats	Cosmos 782	19.5	4	-27.6	[-141.4, 86.3]
	Cosmos 936	18.5	4	-52.6	[-103.8, -1.4]
	Spacelab 3	7	6	-32.0	[-73.9, 9.8]
	Cosmos 2044	14	5	-42.9	[-102.4, 16.5]
**Rats Overall**				**-40.12**	**[-67.72, -12.51]**
				**I^2 = 0**	

Supplementary Table 17. Elastic modulus

Species	Flight	Days	n_{SF}/n_{GC}	SF vs GC ES (%)	SF vs GC 95% CI	GC vs VC ES (%)	GC vs VC 95% CI
Rats	Spacelab 3	7	6	-24.4	[-58.7, 9.9]	*NA*	*NA*
	Cosmos 1887	12.5	4/5	10.4	[-14.4, 35.2]	-18.9	[-46.6, 8.8]
	Cosmos 2044	14	5	27.7	[-2.2, 57.6]	-6.3	[-37.6, 24.9]
	STS-78	17	6/5	-12.4	[-43.0, 18.3]	-27.5	[-45.1, -9.9]
**Rats Overall**				**1.64**	**[-19.98, 23.26]**	**-21.61**	**[-35.02, -8.19]**
				$I^2 = 52.9$		$I^2 = 0$	

Supplementary Table 18. Yield stress

Species	Flight	Days	n_{SF}/n_{GC}	SF vs GC ES (%)	SF vs GC 95% CI	GC vs VC ES (%)	GC vs VC 95% CI
Rats	Spacelab 3	7	6	-14.5	[-40.8, 11.8]	*NA*	*NA*
	Cosmos 1887	12.5	4/5	31.0	[11.0, 50.9]	-27.2	[-46.6, -7.7]
	STS-78	17	6/5	-5.9	[-39.3, 27.5]	-5.6	[-25.0, 13.8]
**Rats Overall**				**4.96**	**[-26.04, 35.97]**	**-16.36**	**[-37.50, 4.78]**
				$I^2 = 76.3$		$I^2 = 57.8$	

Supplementary Table 19. Normalized elastic modulus

Species	Flight	Days	n_{SF}	SF vs GC ES (%)	SF vs GC 95% CI
Rats	Spacelab 3	7	6	-21.4	[-55.7, 13.0]
	Cosmos 1887	12.5	4	27.1	[2.3, 51.9]
	Cosmos 2044	14	5	29.6	[-0.3, 59.5]
	STS-78	17	6	-15.8	[-46.5, 14.9]
**Rats Overall**				**6.21**	**[-20.05, 32.46]**
				$I^2 = 68.0$	

Supplementary Table 20. Normalized yield stress

Species	Flight	Days	n_{SF}	SF vs GC ES (%)	SF vs GC 95% CI
Rats	Spacelab 3	7	6	-11.1	[-37.4, 15.3]
	Cosmos 1887	12.5	4	50.8	[30.9, 70.8]
	STS-78	17	6	-9.6	[-43.0, 23.8]
**Rats Overall**				**11.20**	**[-33.42, 55.83]**
				$I^2 = 88.6$	

Supplementary Table 21. Calcium content

				SF vs GC		GC vs VC	
**Species**	**Flight**	**Days**	**n_{SF}/n_{GC}**	**ES (%)**	**95% CI**	**ES (%)**	**95% CI**
Rats	Cosmos 1129	18.5	5	-3.3	[-9.0, 2.5]	-6.0	[-10.9, -1.1]
	Spacelab 3	7	6	-1.5	[-12.2, 9.5]	*NA*	*NA*
	Cosmos 1887	12.5	4/5	-1.8	[-4.4, 0.8]	-4.6	[-7.7, -1.6]
	Cosmos 2044	14	5/3	5.9	[-6.6, 18.3]	-0.6	[-15.4, 14.2]
	STS-58	14	5	-0.9	[-3.0, 1.2]	*NA*	*NA*
	STS-78	17	6	-2.6	[-4.6, -0.5]	2.3	[-0.7, 5.4]
**Rats Overall**				**-1.75**	**[-2.97, -0.52]**	**-2.40**	**[-7.17, 2.37]**
				**I^2 = 0**		**I^2 = 77.0**	

Supplementary Table 22. Phosphorus content

				SF vs GC		GC vs VC	
**Species**	**Flight**	**Days**	**n_{SF}/n_{GC}**	**ES (%)**	**95% CI**	**ES (%)**	**95% CI**
Rats	Cosmos 1129	18.5	5	-1.8	[-5.0, 1.3]	-9.5	[-12.2, -6.7]
	Spacelab 3	7	6	-0.2	[-6.3, 5.8]	*NA*	*NA*
	Cosmos 1887	12.5	4/5	-2.0	[-8.4, 4.3]	-0.8	[-7.2, 5.5]
	Cosmos 2044	14	5/3	9.9	[-7.8, 27.6]	-4.9	[-24.9, 15.0]
	STS-58	14	5	-1.3	[-4.0, 1.4]	*NA*	*NA*
**Rats Overall**				**-1.32**	**[-3.18, 0.54]**	**-5.71**	**[-12.73, 1.32]**
				**I^2 = 0**		**I^2 = 66.9**	

Supplementary Table 23. Hydroxyproline content

				SF vs GC		GC vs VC	
**Species**	**Flight**	**Days**	**n_{SF}/n_{GC}**	**ES (%)**	**95% CI**	**ES (%)**	**95% CI**
Rats	Spacelab 3	7	6	4.0	[-40.8, 48.7]	*NA*	*NA*
	Cosmos 1887	12.5	4/5	0.0	[-5.4, 5.4]	0.0	[-5.8, 5.8]
	Cosmos 2044	14	5/2	8.8	[-7.9, 25.5]	5.4	[-36.1, 46.9]
**Rats Overall**				**8.20**	**[-7.42, 23.83]**	**0.11**	**[-5.61, 5.84]**
				**I^2 = 0**		**I^2 = 0**	

Supplementary Table 24. Osteocalcin content

				SF vs GC		GC vs VC	
**Species**	**Flight**	**Days**	**n_{SF}/n_{GC}**	**ES (%)**	**95% CI**	**ES (%)**	**95% CI**
Rats	Spacelab 3	7	6	-4.5	[-13.0, 4.0]	*NA*	*NA*
	Cosmos 1887	12	5	4.8	[-8.8, 18.4]	-8.4	[-19.9, 3.1]
	Cosmos 2044	14	4/3	-3.0	[27.3, 21.4]	4.0	[-24.9, 33.0]
**Rats Overall**				**-1.99**	**[-8.90, 4.91]**	**-6.68**	**[-17.38, 4.02]**
				$I^2 = 0$		$I^2 = 0$	

Table legend for Supplementary Tables 6-24:
Days = mission duration; n_{SF} = spaceflight animal group sample size; n_{GC} = ground control sample size (only indicated if differ from SF group). SF vs GC indicates outcomes of spaceflight to ground control comparisons. GC vs VC indicates outcomes of ground control to vivarium control comparisons. ES (%) = effect size or percent difference; 95% CI = 95% confidence interval. *appears next to missions where a GC was not present, and a VC was used as the comparison control for SF vs GC outcomes.

Supplementary Figures

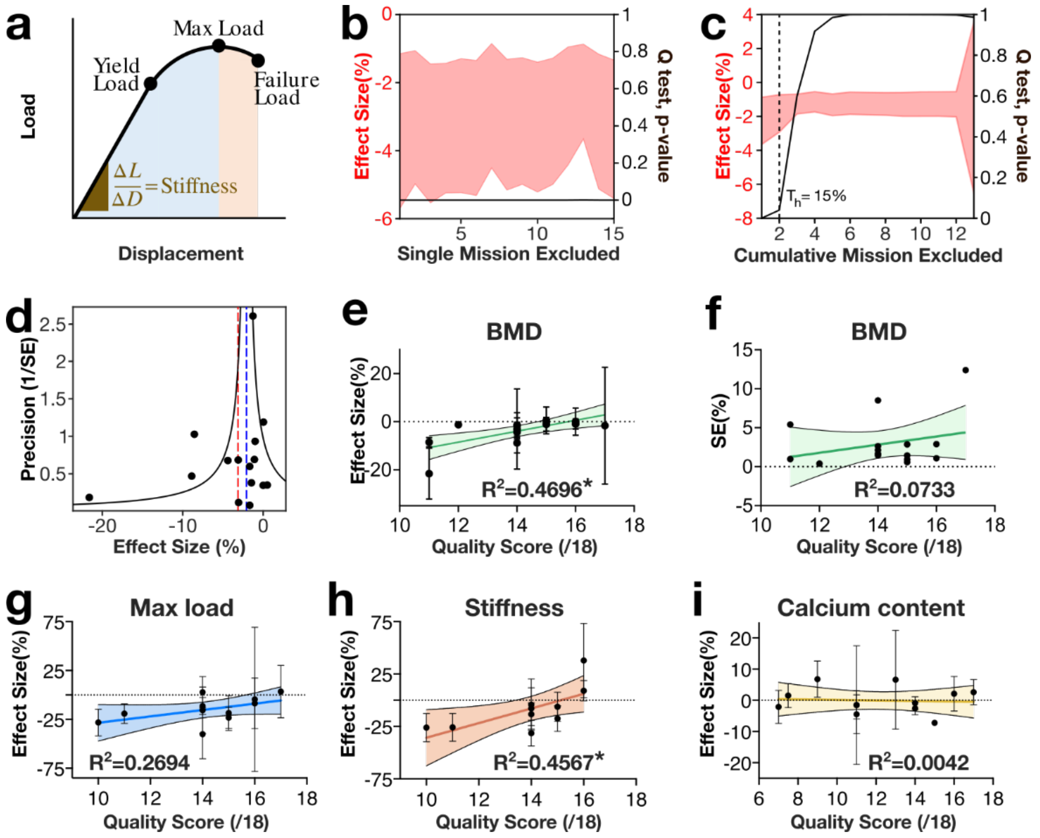

Supplementary Figure 1. Data preparation and appraisal. a) Schematics of load-displacement curve and related parameters selected for study. **b-f)** Bone density dataset was used as the largest in the study, and data appraisal was performed. **b, c)** Single **(b)** and cumulative **(c)** mission exclusion analysis. *Left axis*: 95% CI for global effect size is indicated by the red area. *Right axis*: Q test p-value is indicated by the black line. Homogeneity threshold T_H indicates when Q test p-value ≥ 0.05. **d)** Funnel plot. **e,f)** Meta regression analysis for effect size (ES, %) **(e)**, and standard error (SE, %) **(f)**, as a function of article quality score assessed on an 18 point scale **(Supplementary note 1)**. Meta-regression of article-level effect size (ES, %) of bone max load **(g)**, stiffness **(h)**, and calcium content **(i)** as a function of quality score. Linear regression line (*dark colour*), its 95% CI (*light colour area*) and R^2 are shown.

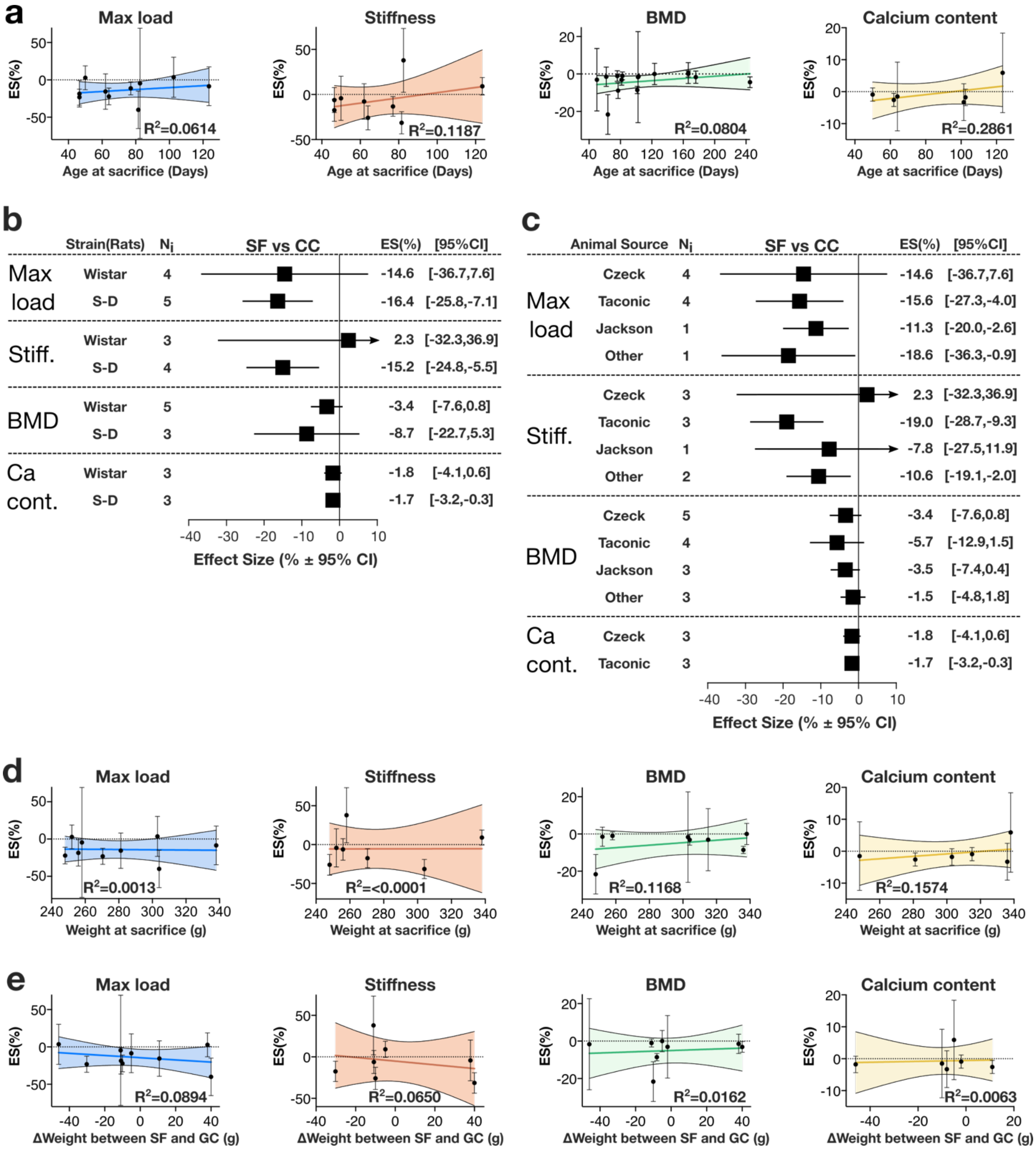

Supplementary Figure 2. Covariate analysis of animal related parameters. a,d,e) Meta-regression analysis of mission-level max load, stiffness, BMD, and calcium content effect size (ES, %), as a function of age of spaceflight rodents at the time of sacrifice **(a)**, rat weight at the time of sacrifice **(d)**, and difference in rat weight between SF and GC **(e)**. Linear regression line (*dark colour*), its 95% CI (*light colour area*) and R^2 are shown. **b,c)** Subgroup analysis of mission-level bone max load, stiffness, BMD, and calcium content by strain of rats, Wistar or Sprague-Dawley (S-D) **(b)**, and source of animals **(c)**, which included: Institute of experimental Endochronology in Czeckolslovakia (Czeck), Taconic Farms in New York (Taconic), Jackson Laboratory; Bar Harbor, ME (Jackson), or other. Square/line: effect size (%) and 95% CI. $N_{i:}$ number of mission level outcomes.

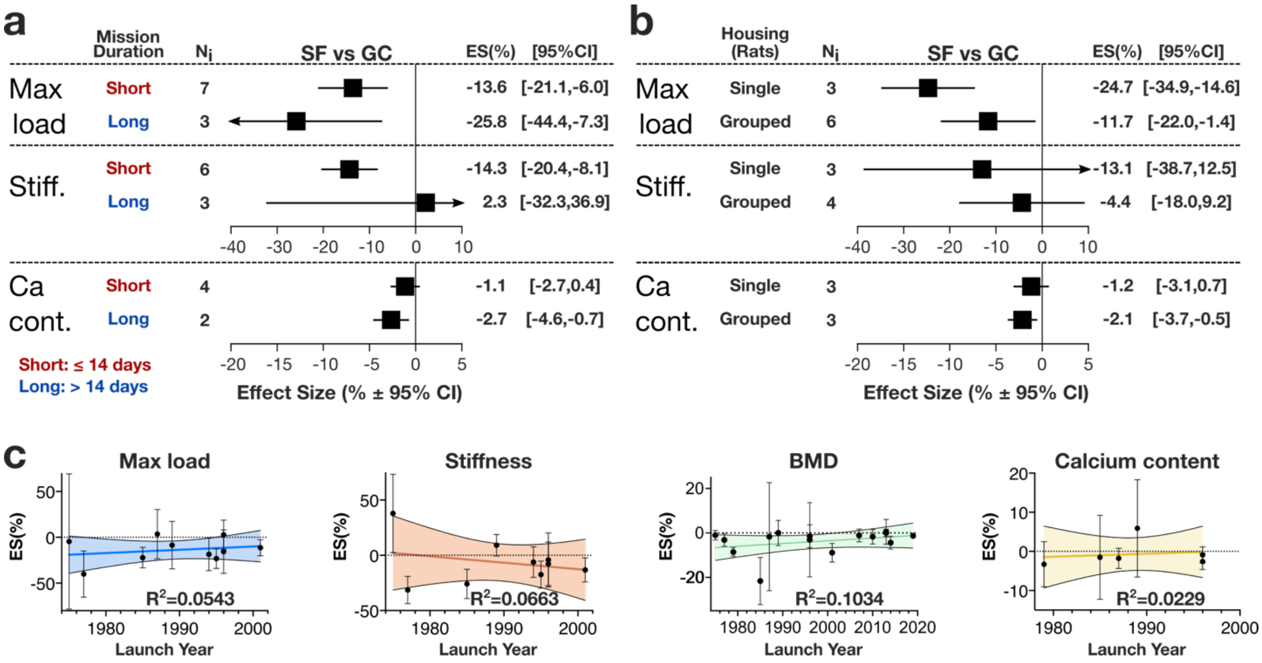

Supplementary Figure 3. Covariate analysis of mission related parameters. a,b) Subgroup analysis of mission-level bone max load, stiffness, and calcium content by mission duration of short (14 days or less) and long (greater than 14 days) missions **(a)**, and single vs. grouped housing for rats **(b)**. Square/line: effect size (%) and 95% CI. $N_{i:}$ number of mission level outcomes. **c)** Meta-regression analysis of mission-level max load, stiffness, BMD, and calcium content effect size (ES, %), as a function of year of mission's launch. Linear regression line (*dark colour*), its 95% CI (*light colour area*) and R^2 are shown.

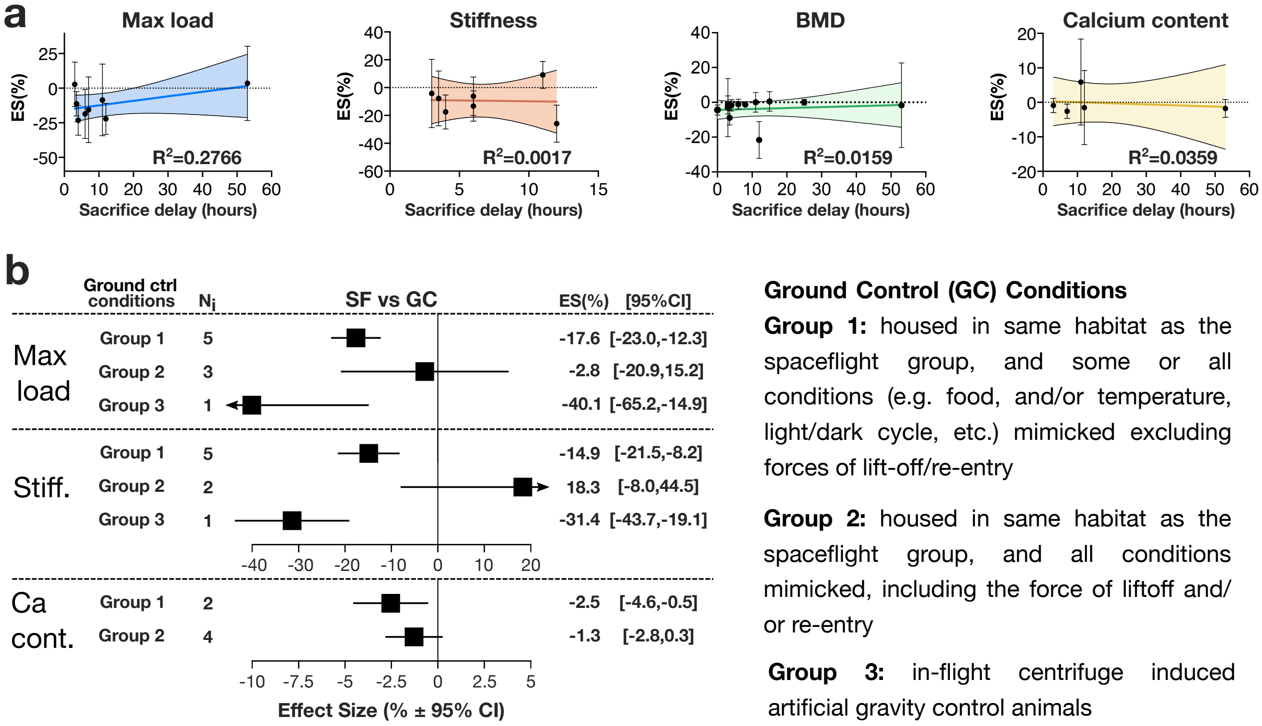

Supplementary Figure 4. Covariate analysis for study related parameters. a) Meta-regression analysis of mission-level max load, stiffness, BMD, and calcium content effect size (ES, %), as a function of delay between space-shuttle landing and time of animal sacrifice (hours). Linear regression line (*dark colour*), its 95% CI (*light colour area*) and R^2 are shown. **b)** Subgroup analysis of mission-level bone max load, stiffness, and calcium content by how closely GC mimics SF conditions: Group 1: housed in same habitat as the spaceflight group, and some or all spaceflight conditions mimicked excluding forces of lift-off/re-entry; Group 2: housed in same habitat as the spaceflight group, and all conditions mimicked, including the force of liftoff and/or re-entry; Group 3: in-flight centrifuge induced artificial gravity control animals. Square/line: effect size (%) and 95% CI. N_i: number of mission level outcomes.

Supplementary References

1 Morey-Holton, E. & Baylink, D. J. Quantitative Analysis of Selected Bone Parameters. Report No. TM-78525, 321-351 (NASA, 1978).

2 Morey-Holton, E., Turner, R. T. & Baylink, D. J. Quantitative Analysis of Selected Bone Parameters. Report No. TM-78526, 135-178 (NASA, 1978).

3 Prokhonchukov, A. A., Desiatnichenko, K. S., Tigranian, R. A. & Komissarova, N. A. [Mineral phase and protein matrix status of rat bony tissue after a flight on the Kosmos-1129 biosatellite]. *Kosm Biol Aviakosm Med* **16**, 61-64 (1982).

4 Rogacheva, I. V., Stupakov, G. P., Volozhin, A. I., Pavlova, M. N. & Poliakov, A. N. [Characteristics of bone tissue of rats after flight aboard biosputnik Kosmos-1129]. *Kosm Biol Aviakosm Med* **18**, 39-44 (1984).

5 Patterson-Buckendahl, P. E., Grindeland, R. E., Martin, R. B., Cann, C. E. & Arnaud, S. B. Osteocalcin as an indicator of bone metabolism during spaceflight. *Physiologist* **28**, S227-S228 (1985).

6 Patterson-Buckendahl, P. *et al.* Fragility and composition of growing rat bone after one week in spaceflight. *Am. J. Physiol.* **252**, R240-246 (1987).

7 Shaw, S. R., Vailas, A. C., Grindeland, R. E. & Zernicke, R. F. Effects of a 1-wk spaceflight on morphological and mechanical properties of growing bone. *Am. J. Physiol.* **254**, R78-83 (1988).

8 Simmons, D. J., Russell, J. E. & Grynpas, M. D. Bone maturation and quality of bone material in rats flown on the space shuttle 'Spacelab-3 Mission'. *Bone Miner.* **1**, 485-493 (1986).

9 Cann, C., Rakhmanov, A. & Karolkov, V. Analysis of Radiographs and Biosamples From Primate Studies. Report No. TM-102254, 513-519 (NASA, 1990).

10 Simmons, D. J., Grynpas, M. D. & Rosenberg, G. D. Maturation of bone and dentin matrices in rats flown on the Soviet biosatellite Cosmos 1887. *FASEB J.* **4**, 29-33 (1990).

11 Vailas, A. C. *et al.* Effects of spaceflight on rat humerus geometry, biomechanics, and biochemistry. *FASEB J.* **4**, 47-54 (1990).

12 Arnaud, S. B., Fung, P., Popova, I. A., Morey-Holton, E. R. & Grindeland, R. E. Circulating parathyroid hormone and calcitonin in rats after spaceflight. *J. Appl. Physiol.* **73**, 169s-173s (1992).

13 Cann, C. E., Patterson-Buckendahl, P., Durnova, G. & Kaplansky, A. Mineral Distribution and Balance in Rats During Spaceflight. Report No. TM-108802, 225-233 (NASA, 1994).

14 Vailas, A. C. *et al.* Adaptation of young adult rat cortical bone to 14 days of spaceflight. *J. Appl. Physiol.* **73**, 4S-9S (1992).

15 Vailas, A. C. *et al.* Biomechanical, Biochemical, and Morphological Alterations of Intramuscular and Dense Fibrous Connective Tissues After 14 Days Spaceflight: I. Connective Tissue Studies. Report No. TM-108802, 103-152 (NASA, 1994).

16 Lafage-Proust, M. H. *et al.* Space-related bone mineral redistribution and lack of bone mass recovery after reambulation in young rats. *Am. J. Physiol.* **274**, R324-334 (1998).

17 Chapes, S. K., Simske, S. J., Sonnenfeld, G., Miller, E. S. & Zimmerman, R. J. Effects of spaceflight and PEG-IL-2 on rat physiological and immunological responses. *Journal of Applied Physiology* **86**, 2065-2076 (1999).

18 Bateman, T. A. *et al.* Histomorphometric, physical, and mechanical effects of spaceflight and insulin-like growth factor-I on rat long bones. *Bone* **23**, 527-535 (1998).
19 Vajda, E. G., Wronski, T. J., Halloran, B. P., Bachus, K. N. & Miller, S. C. Spaceflight alters bone mechanics and modeling drifts in growing rats. *Aviat. Space Environ. Med.* **72**, 720-726 (2001).
20 Zerath, E. *et al.* Spaceflight inhibits bone formation independent of corticosteroid status in growing rats. *J. Bone Miner. Res.* **15**, 1310-1320 (2000).
21 Lloyd, S. A. *et al.* Osteoprotegerin is an effective countermeasure for spaceflight-induced bone loss in mice. *Bone* **81**, 562-572 (2015).
22 Ortega, A. M. *et al.* in *ASME 2013 Summer Bioengineering Conference* Vol. 1A: Abdominal Aortic Aneurysms; Active and Reactive Soft Matter; Atherosclerosis; BioFluid Mechanics; Education; Biotransport Phenomena; Bone, Joint and Spine Mechanics; Brain Injury; Cardiac Mechanics; Cardiovascular Devices, Fluids and Imaging; Cartilage and Disc Mechanics; Cell and Tissue Engineering; Cerebral Aneurysms; Computational Biofluid Dynamics; Device Design, Human Dynamics, and Rehabilitation; Drug Delivery and Disease Treatment; Engineered Cellular Environments (Sunriver, Oregon, USA, 2013).
23 Coulombe, J. C. *et al.* Microgravity-induced alterations of mouse bones are compartment- and site-specific and vary with age. *Bone* **151**, 116021, doi:10.1016/j.bone.2021.116021 (2021).
24 Zhang, B., Cory, E., Bhattacharya, R., Sah, R. & Hargens, A. R. Fifteen days of microgravity causes growth in calvaria of mice. *Bone* **56**, 290-295 (2013).
25 Gerbaix, M. *et al.* One-month spaceflight compromises the bone microstructure, tissue-level mechanical properties, osteocyte survival and lacunae volume in mature mice skeletons. *Sci. Rep.* **7**, 2659 (2017).
26 Gerbaix, M. *et al.* Eight Days of Earth Reambulation Worsen Bone Loss Induced by 1-Month Spaceflight in the Major Weight-Bearing Ankle Bones of Mature Mice. *Front. Physiol.* **9**, 746 (2018).
27 Macaulay, T. R., Siamwala, J. H., Hargens, A. R. & Macias, B. R. Thirty days of spaceflight does not alter murine calvariae structure despite increased Sost expression. *Bone Rep.* **7**, 57-62 (2017).
28 Lee, S.-J. *et al.* Targeting myostatin/activin A protects against skeletal muscle and bone loss during spaceflight. *Proc. Natl. Acad. Sci. U.S.A.* **117**, 23942-23951 (2020).
29 Fu, J. *et al.* Bone health in spacefaring rodents and primates: systematic review and meta-analysis. *NPJ Microgravity* **7**, 19 (2021).
30 Mack, P. B. Bone density changes in a Macaca nemestrina monkey during the biosatellite 3 project. *Aerosp. Med.* **42**, 828-833 (1971).
31 Asling, C. W. Histological Studies on Tibial Bone of Rats in the 1975 Cosmos-782 Flight: I. Endochondral Osteogenesis; Medullary Bone Turnover. Report No. TM-78525, 276-290 (NASA, 1978).
32 Spengler, D. M., Morey, E. R., Carter, D. R., Turner, R. T. & Baylink, D. J. Effect of space flight on bone strength. *Physiologist* **22**, S75-76 (1979).
33 Spengler, D. M., Morey, E. R., Carter, D. R., Turner, R. T. & Baylink, D. J. Effects of Spaceflight on Structural and Material Strength of Growing Bone. *Exp. Biol. Med. (Maywood)* **174**, 224-228 (1983).

34 Kazarian, L. E. Vertebral Body Stength of Rat Spinal Columns. 229-266 (NASA, 1981).
35 Kazarian, L. E., Collins, G., Muhic, L. & Becton, F. in *Gravitational Physiology* (eds J. Hideg & O. Gazenko) 129-138 (Pergamon, 1981).
36 Eurell, J. A. & Kazarian, L. E. Quantitative histochemistry of rat lumbar vertebrae following spaceflight. *Am. J. Physiol.* **244**, R315-318 (1983).
37 France, E. P., Oloff, C. M. & Kazarian, L. E. Bone Mineral Analysis of Rat Vertebrae Following Space Flight: COSMOS 1129. Report No. AFAMRL-TR-83-055, 1-27 (Air Force Aerospace Medical Research Lab, 1983).
38 Russell, J. E. & Simmons, D. J. Bone maturation in rats flown on the Spacelab-3 mission. *Physiologist* **28**, S235-236 (1985).
39 Arnaud, S. *et al.* Distribtuion and Biochemistry of Mineral and Matrix in the Femurs of Rats. Report No. TM-102254, 63-84 (NASA, 1990).
40 Mechanic, G. L. *et al.* Regional distribution of mineral and matrix in the femurs of rats flown on Cosmos 1887 biosatellite. *FASEB J.* **4**, 34-40 (1990).
41 Simmons, D., Grynpas, M., Rosenberg, G. & Durnova, G. Biomedical Biochemica and morphological alternations of muscle and dense, fibrous connective tissues during 14 days of Spaceflight. Report No. TM-102254, 157-168 (NASA, 1990).
42 Vailas, A., Zernicke, R., Grindeland, R. & Kaplansky, A. Biomedical Biochemica and morphological alternations of muscle and dense, fibrous connective tissues during 14 days of Spaceflight. Report No. TM-102254, 85-112 (NASA, 1990).
43 Zernicke, R. F. *et al.* Spaceflight effects on biomechanical and biochemical properties of rat vertebrae. *Am. J. Physiol. Regul. Integr. Comp. Physiol.* **258**, R1327-R1332 (1990).
44 Rakhmanov, A. S. *et al.* [The state of bone tissue in monkeys in experiments in the Cosmos-1887 biosatellite]. *Kosm Biol Aviakosm Med* **25**, 42-44 (1991).
45 Arnaud, S. *et al.* Bone Biochemistry Mineral Distribution and Calcium Regulating Hormones in Rats After the Cosmos 2044 Biosatellite Flight: I. Circulating Parathyroid Hormone and Calcitonin in Rats after Spaceflight. . Report No. TM-108802, 69-101 (NASA, 1994).
46 Sinha, R. K., Shah, S. A., Hume, E. L. & Tuan, R. S. The effect of a 5-day space flight on the immature rat spine. *Spine J.* **2**, 239-243 (2002).
47 Zerath, E. *et al.* Effects of spaceflight on bone mineralization in the rhesus monkey. *J. Appl. Physiol.* **81**, 194-200 (1996).
48 Arnaud, S. B., Hutchinson, T., Bakulin, A. V. & R., S. C. Bending stiffness of the tibia in young rhesus monkeys after two weeks in space aboard the Cosmos 2229 Biosatellite. Report No. TM-110439, 67-80 (NASA, 1997).
49 Yamada, G. *et al.* Trace element composition and histological analysis of rat bones from the space shuttle. *Life Sci.* **60**, 635-642 (1997).
50 Cavolina, J. *et al.* The Effects of Orbital Spaceflight on Bone Histomorphometry and Messenger Ribonucleic Acid Levels for Bone Matrix Proteins and Skeletal Signaling Peptides in Ovariectomized Growing Rats 1. *Endocrinology* **138**, 1567-1576 (1997).
51 Hatton, D. C. *et al.* Calcium metabolism and cardiovascular function after spaceflight. *J. Appl. Physiol.* **92**, 3-12 (2002).

52 Bailey, J. F., Hargens, A. R., Cheng, K. K. & Lotz, J. C. Effect of microgravity on the biomechanical properties of lumbar and caudal intervertebral discs in mice. *J Biomech* **47**, 2983-2988 (2014).
53 Berg-Johansen, B. *et al.* Spaceflight-induced bone loss alters failure mode and reduces bending strength in murine spinal segments. *J. Orthop. Res.* **34**, 48-57 (2016).
54 Chakraborty, N. *et al.* Gene-metabolite networks associated with impediment of bone fracture repair in spaceflight. *Computational and Structural Biotechnology Journal* **19**, 3507-3520 (2021).
55 Tominari, T. *et al.* Hypergravity and microgravity exhibited reversal effects on the bone and muscle mass in mice. *Sci. Rep.* **9**, 6614 (2019).
